# Metabolic Health and Heterogenous Outcomes of Prenatal Interventions: A Secondary Analysis of a Randomized Clinical Trial

**DOI:** 10.1001/jamanetworkopen.2025.28264

**Published:** 2025-08-21

**Authors:** Emily W. Flanagan, Kimberly L. Drews, W. Todd Cade, Paul W. Franks, Dympna Gallagher, Suzanne Phelan, Linda Van Horn, Leanne M. Redman

**Affiliations:** 1 Pennington Biomedical Research Center, Baton Rouge, Louisiana; 2 Doctor of Physical Therapy Division, Duke University School of Medicine, Durham, North Carolina; 3 Department of Nutrition, Harvard T.H. Chan Public Health School, Harvard University, Boston, Massachusetts; 4 Department of Clinical Sciences, Genetic and Molecular Epidemiology Unit, Lund University Diabetes Centre, Skåne University Hospital Malmö, Malmö, Sweden; 5 New York Obesity Research Center, Department of Medicine, College of Physicians and Surgeons, Columbia University, New York; 6 Institute of Human Nutrition, College of Physicians and Surgeons, Columbia University, New York, New York; 7 Department of Kinesiology and Public Health, California Polytechnic State University, San Luis Obispo; 8 Department of Preventive Medicine, Feinberg School of Medicine, Northwestern University, Chicago, Illinois

## Abstract

**Importance:**

Prenatal intensive behavioral therapy (IBT) interventions that promote adequate gestational weight gain (GWG) have had variable and mostly modest effects on clinically relevant maternal and infant outcomes. It is unknown whether different maternal obesity metabolic phenotypes underlie the heterogeneity in response.

**Objective:**

To examine GWG, adverse perinatal outcomes, substrate changes, and differential changes in each in a prenatal IBT intervention conducted among pregnant individuals with 2 identified obesity phenotypes.

**Design, Setting, and Participants:**

The Lifestyle Interventions for Expectant Moms (LIFE-Moms) trial was a consortium of 7 independent but collaborative multicenter randomized clinical trials that took place between November 1, 2012, and December 31, 2017, and evaluated a prenatal IBT intervention on GWG and perinatal outcomes among women with overweight and obesity. Statistical analysis for the present preplanned secondary analysis was conducted from March 29, 2023, to June 4, 2025. Participants (n = 1150) had a confirmed viable singleton pregnancy and no previous diagnosis of cardiometabolic diseases. This analysis was limited to those with obesity. Participants were classified into 2 groups: obesity with no additional qualifying cardiometabolic disease risk factors (metabolically healthy obesity [MHO]) or obesity with 2 qualifying risk factors (metabolically unhealthy obesity [MUO]) in early pregnancy.

**Intervention:**

A behavioral lifestyle intervention incorporating diet and physical activity was delivered to increase adherence to the National Academy of Medicine GWG guidelines.

**Main Outcomes and Measures:**

GWG (total and adherence to guidelines), adverse perinatal outcomes, substrate changes, and infant body composition. Analysis was conducted on an intent-to-treat basis.

**Results:**

The mean (SD) age of the 640 participants was 30.2 (5.6) years, and the participants presented in early pregnancy with a mean (SD) body mass index of 35.2 (4.1). Participants in the MUO (n = 172) and MHO (n = 228) groups did not differ in response to treatment in weight outcomes, adverse perinatal outcomes, or substrate changes, with the exception that patients in the intervention group experienced smaller mean (SE) triglyceride increases more in the MUO group than in the MHO group (90.3% [7.4%] vs 81.8% [8.3%]; *P* = .02). After adjustment for maternal baseline demographics and treatment group, individuals with MUO had lower GWG (0.30 [0.23] kg/wk) compared with individuals with MHO (0.41 [0.27] kg/wk; *P* < .001), a 36.7% difference, and had a lower proportion exceed weight gain guidelines (57.0% [98 of 172] vs 68.0% [155 of 228]; *P* = .03). Participants with MUO had a higher incidence of gestational diabetes (23.8% [41 of 172] vs 9.8% [22 of 228]; *P* = .001) and had infants with a higher proportion of adiposity (mean [SD], 12.5% [3.9%] vs 11.7% [3.7%] fat; *P* = .05) compared with participants with MHO.

**Conclusions and Relevance:**

In this preplanned secondary analysis of a randomized clinical trial of an IBT on GWG among pregnant individuals, those with MUO experienced less GWG, had a higher incidence of gestational diabetes, and had infants with a higher proportion of adiposity compared with those who MHO. Data supported that the maternal obesity metabolic phenotype has a greater clinical effect on adverse maternal and infant clinical outcomes compared with GWG alone and did not contribute to a differential response to a lifestyle intervention. Future interventions should identify methods to better optimize the maternal metabolic milieu as early in pregnancy as possible to reduce the intergenerational transmission of metabolic disease.

**Trial Registration:**

ClinicalTrials.gov Identifiers: NCT01545934, NCT01616147, NCT01771133, NCT01631747, NCT01768793, NCT01610752, NCT01812694

## Key Points

**Question:**

Do preexisting metabolic phenotypes of obesity differentially influence the outcomes of a prenatal lifestyle intervention aimed at reducing excess gestational weight gain?

**Findings:**

This preplanned secondary analysis of 640 participants in a multisite randomized clinical trial showed that pregnant individuals with a metabolically unhealthy obesity phenotype had 36.7% lower gestational weight gain but a higher incidence of gestational diabetes and infants born with greater adiposity compared with pregnant individuals with obesity and a healthier metabolic phenotype in early pregnancy. Participants in both phenotype groups responded similarly to a prenatal lifestyle intervention.

**Meaning:**

Gestational weight gain may be less important than the maternal metabolic milieu in estimating perinatal outcomes and infant health, highlighting the need for prenatal interventions that focus on optimizing maternal metabolic health.

## Teaser

This preplanned secondary analysis of a randomized clinical trial estimates the effect of maternal obesity phenotype on gestational weight gain, adverse perinatal outcomes, substrate changes, and infant body composition for participants in a prenatal intensive behavioral therapy intervention.

## Introduction

The intergenerational transmission of obesity is modifiable and may serve as a potential mechanism to reduce the increasing obesity rate. Prepregnancy obesity and excess gestational weight gain (GWG) are both risk factors for maternal morbidity and adverse offspring outcomes, including infant size and adiposity at birth.^1^ The National Academies of Science, Engineering, and Medicine (NAM; previously the Institute of Medicine [IOM]) recommend a range of weight gain dependent on body mass index (BMI; calculated as weight in kilograms divided by height in meters squared) to minimize adverse maternal, pregnancy, and infant outcomes. However, only one-third of women achieve the recommended weight gain during pregnancy. As such, the US Preventive Services Task Force (USPSTF) recommends prenatal lifestyle interventions for all pregnant individuals to manage GWG.^2^

Numerous randomized clinical trials have been successfully executed and demonstrate a reduction in GWG. Such intensive behavioral therapy (IBT) interventions target modifications to diet and/or physical activity, with the end goal of altering energy balance to attenuate GWG. Large systematic reviews show that the mean GWG difference between those who receive lifestyle interventions and those who receive usual care during pregnancy is approximately 1.2 kg, with large heterogeneity in both maternal and offspring outcomes.^3,4,5,6^ For these reasons, it is worthwhile to evaluate identified pregnancy phenotypes as a mechanism for explaining differences in maternal and offspring outcomes in response to lifestyle interventions.

One such identified phenotype is in pregnant individuals with obesity and involves differences in the early metabolic milieu. Pregnant individuals with obesity plus additional underlying metabolic risk factors (ie, metabolically unhealthy obesity [MUO] phenotype) have elevated circulating growth-promoting substrates (ie, glucose and lipids) throughout pregnancy and demonstrate greater fetal adipose tissue accretion compared with their counterparts with obesity but without additional metabolic risks (ie, metabolically healthy obesity [MHO] phenotype).^7,8^ However, in an observational cohort, differences in GWG—a major modifiable risk factor for adverse perinatal outcomes—were not apparent between phenotypes. It is unknown how pregnant individuals with this identified obesity phenotype (ie, MUO vs MHO) respond to a prenatal lifestyle intervention aimed at reducing GWG. We hypothesized that differences in the obesity phenotype may underlie the differential responses in maternal substrate changes, adverse perinatal outcomes, and infant size and adiposity to a prenatal lifestyle intervention, thus diluting the beneficial outcomes of rigorous trials. Understanding the association between early pregnancy metabolic health and downstream outcomes in a large randomized clinical trial with a diverse sample may be integral to understanding the effects of prenatal lifestyle interventions and identifying the participants who may not optimally respond. Therefore, it is important to evaluate responses to prenatal interventions by obesity phenotypes to understand the effect of existing prenatal GWG trials.

The Lifestyle Interventions for Expectant Moms (LIFE-Moms) trial was a consortium of 7 independent but collaborative clinical trials to evaluate the efficacy of prenatal IBT interventions on GWG and maternal and offspring outcomes among women with overweight and obesity. These trials showed that prenatal lifestyle interventions reduced GWG by 1.6 kg yet, in line with similar trials, failed to demonstrate an improvement to downstream maternal and neonatal outcomes.^9^ The aims of the present analysis were to identify (1) the differences in GWG and adherence to NAM guidelines between pregnant individuals with a MHO phenotype and those with a MUO phenotype, (2) the incidence of perinatal adverse outcomes and infant health between the 2 identified obesity phenotypes, and (3) differential responses to a prenatal IBT intervention.

## Methods

### Study Design

This is a preplanned secondary analysis of a multisite randomized clinical trial, LIFE-Moms. The LIFE-Moms trial has been previously described (trial protocol and statistical analysis plan in Supplement 1).^9,10^ In brief, LIFE-Moms was a research consortium initiated by the National Institutes of Health to reduce excessive GWG in individuals with overweight and obesity. The consortium included 7 clinical sites, a research coordinating unit, and National Institutes of Health–sponsored institutes and centers. Each of the 7 clinical sites conducted an independent and innovative randomized clinical trial to evaluate the effects of an IBT intervention incorporating dietary and/or physical activity components on improving the incidence of appropriate GWG based on the 2009 NAM guidelines (eTable 1 in Supplement 2).^11,12,13,14,15,16^ The randomized clinical trials took place between November 1, 2012, and December 31, 2017. Eligibility criteria for the trial included a singleton pregnancy less than 15 weeks and 6 days of gestation, age 18 years or older, and BMI of 25 or more at the time of screening. Exclusion criteria included a diagnosis of diabetes prior to pregnancy or a study-assessed hemoglobin A_1c_ (HbA_1c_) level of 6.5% or more at screening, known fetal anomaly prior to enrollment, history of 3 of more consecutive first-trimester miscarriages, current diagnosed eating disorder, prior or planned bariatric surgery, and/or use of exclusionary medications (metformin, systemic corticosteroids, antipsychotic agents, antiseizure medications or mood stabilizers that would be expected to have a significant effect on body weight, amphetamines, and methylphenidate). A total of 1150 pregnant individuals with overweight or obesity were enrolled across 7 sites. Although interventions differed across sites, the centers standardized time points and outcomes of interest. Pregnancy outcomes were obtained by blinded assessors in early (9-16 weeks’ gestation), middle (24-28 weeks’ gestation), and late (35-36 weeks’ gestation) pregnancy. Randomization occurred after the early pregnancy visit. Offspring outcomes were obtained between 24 hours and 1 week of life, with the exception of infants born prior to 37 weeks of gestation who were measured once the infant reached 37 weeks. Institutional review boards for each site (California Polytechnic State University, Brown University, St. Luke’s–Roosevelt Hospital, Columbia University, University of Puerto Rico, Northwestern University, Washington University, Pennington Biomedical Research Center, National Institute of Diabetes and Digestive and Kidney Diseases/Phoenix Indian Medical Center) and the LIFE-Moms data safety monitoring board approved and monitored the conduct of the trials and consortium activities. Study participants provided written informed consent prior to participation. The study followed the Consolidated Standards of Reporting Trials (CONSORT) reporting guideline.

### Participants

For the present analysis, only individuals with obesity (BMI ≥30) at study entry were included. Individuals were excluded at screening if they had a diagnosis of diabetes prior to pregnancy or an HbA_1c_ level of 6.5% or more, a known fetal anomaly, a history of 3 or more consecutive first-trimester miscarriages, a history of anorexia or bulimia, a current eating disorder, active suicidal ideation, prior or planned bariatric surgery, current use of exclusionary medications, and contraindications to aerobic exercise in pregnancy.^9^

### Maternal Anthropometrics

Weight was measured in duplicate during early, middle, and late pregnancy, and the mean of the 2 measurements was used. To account for varying lengths of gestation, GWG was calculated as the weight difference between the early and late pregnancy visits, divided by the number of weeks between the 2 visits. Excess GWG was defined as GWG per week above the 2009 IOM recommendations of second- and third-trimester weight gain for pregnant women with obesity (>0.27 kg/wk).^26^

### Cardiometabolic Biomarkers and Metabolic Phenotyping

Maternal substrates were assessed by fasting blood samples obtained in early and late pregnancy. Samples were collected and processed at each clinical site and sent to a core laboratory (Washington University School of Medicine in St Louis, Missouri) for analyses. Glucose, insulin, C-peptide, leptin, adiponectin, tumor necrosis factor, interleukin 6, high-density lipoprotein cholesterol (HDL-C), low-density lipoprotein cholesterol (LDL-C), and triglyceride concentrations were quantified. Blood pressure was obtained in duplicate after a 5-minute seated rest.

The maternal obesity metabolic phenotype was determined using the National Cholesterol Education Program Adult Treatment Panel III guidelines for metabolic risk.^17^ Individuals were classified as having MUO or MHO using the following metabolic risk factors: systolic blood pressure higher than 130 mm Hg or diastolic blood pressure higher than 85 mm Hg, HDL-C level lower than 50 mg/dL (to convert to millimoles per liter, multiply by 0.0259), LDL-C level of 100 mg/dL or higher (to convert to millimoles per liter, multiply by 0.0259), triglyceride level of 150 mg/dL or higher (to convert to millimoles per liter, multiply by 0.0113), and glucose level of 100 mg/dL or higher (to convert to millimoles per liter, multiply by 0.0555). For MHO, obesity presented with no aforementioned risk factors in early pregnancy. Conversely, for MUO, obesity presented with 2 or more aforementioned risk factors. To strengthen our approach to defining 2 distinct and dichotomous groups, individuals with obesity and only 1 risk factor were excluded from analysis.

### Maternal, Pregnancy, and Neonatal Adverse Outcomes

The maternal delivery and neonatal medical record were abstracted after delivery. Records were abstracted for delivery route, pregnancy complications, maternal diagnosis of gestational diabetes, preeclampsia or gestational hypertension, and neonatal health outcomes.^7,8,9^

### Offspring Anthropometrics

Infant weight and length were obtained in duplicate. Skinfold thicknesses were obtained on the triceps, subscapular, iliac crest, and thigh. Sum of skinfolds was calculated, and fat mass was estimated.^18,19^ Infant fat mass index was calculated as fat mass divided by infant length.^2^^,^^20^

### Statistical Analysis

Statistical analysis was conducted on an intent-to-treat basis from March 29, 2023, to June 4, 2025. Descriptive statistics are reported as mean (SD) values for continuous variables and percentages for categorical variables with 95% CIs both overall and by metabolic health category. Analysis of continuous outcome measures, with outcome variables log transformed to obtain approximate normality as needed, were conducted using general linear mixed models, while analysis of categorical variable outcomes used a generalized linear mixed model. Both model types included a random effect for individual study, due to the nature of the combined LIFE-Moms trial analysis. Demographic characteristics at baseline between the groups were compared without covariate adjustment, while baseline cardiometabolic profile variables were adjusted for BMI at enrollment, self-reported race and ethnicity (Hispanic, non-Hispanic African American, non-Hispanic White, and other [American Indian or Native Alaskan, Asian, and Native Hawaiian or Other Pacific Islander] or mixed), and maternal age at enrollment. Centers for Disease Control and Prevention race and ethnicity categories were used. We assessed race and ethnicity because the literature supports known differences in perinatal risk factors among certain racial and ethnic groups. All pregnancy, delivery, and infant outcomes were modeled both without and with covariate adjustments. For models after baseline, because all participants were included regardless of randomization assignment, treatment was included as a covariate. Models for maternal outcomes also included maternal age, race and ethnicity, and BMI as covariates. Gestational age at delivery, infant sex, and infant age were additional covariates added to the models for infant outcomes. To assess the effect of the IBT on the maternal and infant outcomes, an interaction term for treatment with obesity metabolic phenotype was added into the model. For all outcomes, a nominal 2-sided *P* ≤ .05 was considered to indicate statistical significance; because this was a secondary data analysis, *P* values were not adjusted for multiple testing. Analyses were performed using SAS, version 9.4 (SAS Institute Inc).

## Results

### Participants

**Figure 1.**
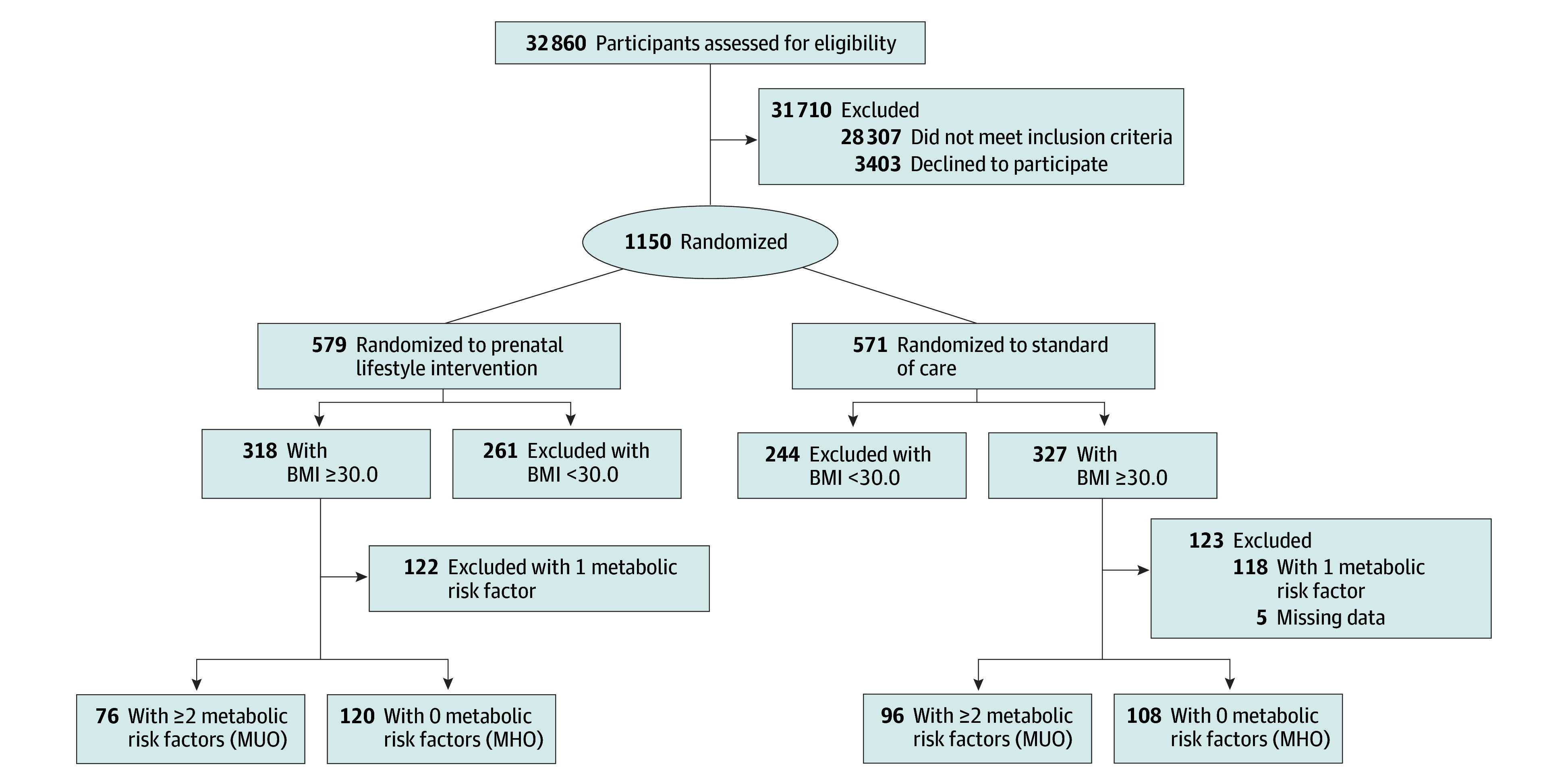
Flow Diagram BMI indicates body mass index (calculated as weight in kilograms divided by height in meters squared); MHO indicates metabolically healthy obesity; and MUO, metabolically unhealthy obesity.

In total, 640 of 1150 individuals (55.7%) had obesity in early pregnancy and complete phenotypic data. Participants with obesity had a mean (SD) age of 30.2 (5.6) years and presented in early pregnancy with a mean (SD) BMI of 35.2 (4.1). Of those, 228 (35.6%) had obesity with no additional measured metabolic risk factors (ie, MHO), 172 (26.9%) had obesity with 2 or more additional measured metabolic risk factors (ie, MUO), and 240 (37.5%) had obesity with 1 additional measured metabolic risk factor and were therefore excluded from analyses (Figure 1). Of those with MUO, 124 had 2 additional risk factors, 35 had 3 additional risk factors, 12 had 4 additional risk factors, and 1 had 5 additional risk factors (eFigure 1A in Supplement 2). Individuals with MHO were significantly younger than those with MUO and had a lower BMI at baseline (Table 1). Metabolic phenotypes additionally differed by race and ethnicity. No differences were observed in income, educational level, or parity. Randomization assignment was similar between obesity metabolic phenotypes: 47.4% (108 of 228) participants with MHO were assigned to receive an IBT intervention compared with 55.8% (96 of 172) participants with MUO (*P* = .10).

**Table 1.** Baseline Characteristics and Cardiometabolic Risk Profile^a^

Characteristic	Mean (95% CI) [range]			*P* value
	Overall (N = 400)	Metabolically healthy obesity (n = 228)	Metabolically unhealthy obesity (n = 172)	
Maternal age at randomization, y	30.2 (29.7-30.8) [18.4-48.7]	29.7 (29.0-30.4) [18.6-43.5]	30.9 (30.1-31.8) [18.4-48.7]	.03
Gestational age at randomization, wk	13.6 (13.4-13.7) [9.1-16.4]	13.3 (13.1-13.6) [9.1-15.9]	13.9 (13.6-14.1) [9.6-16.4]	.003
BMI	35.2 (34.8-35.6) [30.0-51.2]	34.8 (34.3-35.3) [30.0-46.71]	35.8 (35.1-36.5) [30.1-51.2]	.03
Weight, kg	94.3 (92.9-95.6) [64.8-146.7]	93.3 (91.7-95.0) [71.4-141.2]	95.5 (93.2-97.8) [64.8-146.7]	.12
Race and ethnicity, %				
Hispanic	26.5 (22.2-30.8)	20.2 (14.9-25.4)	34.9 (27.7-42.1)	.003
Non-Hispanic African American	34.3 (29.6-38.9)	39.9 (33.5-46.3)	26.7 (20.0-33.4)	
Non-Hispanic White	32.0 (27.4-36.6)	31.1 (25.1-37.2)	33.1 (26.0-40.2)	
Other or mixed^b^	7.3 (4.7-9.8)	8.8 (5.1-12.5)	5.2 (1.9-8.6)	
Income level, %				
<$25 000	40.7 (35.8-45.5)	42.7 (36.2-49.2)	37.9 (30.5-45.3)	.68
$25 000-$74 999	29.3 (24.8-33.8)	24.2 (18.6-29.8)	36.1 (28.8-43.4)	
≥$75 000	30.1 (25.5-34.6)	33.0 (26.9-39.2)	26.0 (19.4-32.7)	
Maternal education of college or higher, %	42.3 (37.4-47.1)	43.0 (36.5-49.5)	41.3 (33.8-48.7)	.76
Nulliparous, %	36.0 (31.3-40.7)	39.5 (33.1-45.9)	31.4 (24.4-38.4)	.10
Randomization, %				
Intervention	51.0 (46.1-55.9)	47.4 (40.8-53.9)	55.8 (48.3-63.3)	
Control	49.0 (44.1-53.9)	52.6 (46.1-59.2)	44.2 (26.7-51.7)	
Cardiometabolic profile				
Hemoglobin A_1c_, %	5.3 (5.3-5.4) [4.4-6.3]	5.2 (5.2-5.3) [4.4-5.7]	5.5 (5.4-5.5) [4.6-6.3]	<.001
Insulin, µIU/mL	0.2 (0.2-0.2) [0.0-0.8]	0.1 (0.1-0.2) [0.0-0.6]	0.2 (0.2-0.2) [0.0-0.8]	<.001
Systolic blood pressure, mm Hg	110.5 (109.4-111.6) [80.5-152.0]	108.4 (107.2-109.7) [80.5-130.0]	113.2 (111.4-115.0) [88.0-152.0]	<.001
Diastolic blood pressure, mm Hg	66.0 (65.2-66.8) [46.0-98.0]	65.3 (64.3-66.3) [47.7-85.0]	66.9 (65.5-68.2) [46.0-98.0]	.06
Total cholesterol, mg/dL	179.2 (175.8-182.6) [95.0-302.0]	165.6 (162.7-168.5) [120.0-247.0]	196.8 (190.9-202.7) [95.0-302.0]	<.001
Triglycerides, mg/dL^c^	122.1 (117.2-127.0) [42.0-305.0]	95.1 (91.8-98.4) [42.0-150.0]	157.2 (149.4-164.9) [50.0-305.0]	<.001
HDL-C, mg/dL	62.7 (61.2-64.2) [31.0-139.0]	68.8 (67.0-70.6) [50.0-139.0]	54.8 (52.8-56.9) [31.0-107.0]	<.001
LDL-C, mg/dL	92.1 (89.3-94.9) [19.0-203.0]	77.8 (75.6-80.0) [19.0-100.0]	110.5 (106.0-115.0) [34.0-203.0]	<.001
Glucose, mg/dL	88.6 (87.8-89.3) [69.0-127.0]	86.2 (85.4-86.9) [69.0-99.0]	91.6 (90.3-92.9) [76.0-127.0]	<.001
Leptin, µg/L	63.9 (61.5-66.3) [22.9-180.7]	62.8 (59.6-66.0) [22.9-169.8]	65.3 (61.6-69.1) [28.3-180.7]	.15
Adiponectin, ng/mL	3185.8 (2976.8-3394.8) [336.0-14 416.0]	3661.3 (3334.8-3987.9) [336.0-14 416.0]	2566.5 (2374.2-2758.8) [338.0-7491.0]	<.001
TNF, pg/mL	1.1 (1.0-1.2) [0.3-16.5]	1.1 (0.9-1.2) [0.3-16.5]	1.2 (1.1-1.2) [0.3-2.8]	.30
Interleukin-6, pg/mL	2.0 (1.7-2.3) [0.4-59.5]	2.0 (1.5-2.6) [0.4-59.5]	2.0 (1.8-2.1) [0.6-9.1]	.86
C-peptide, ng/mL^c^	2.4 (2.3-2.5) [0.9-6.3]	2.1 (2.0-2.2) [0.9-5.0]	2.7 (2.6-2.9) [1.2-6.3]	<.001
HOMA score^c^	3.7 (3.4-3.9) [0.5-21.2]	3.0 (2.8-3.3) [0.5-15.2]	4.5 (4.1-4.9) [0.7-21.2]	<.001

Abbreviations: BMI, body mass index (calculated as weight in kilograms divided by height in meters squared); HDL-C, high-density lipoprotein cholesterol; HOMA, homeostatic model assessment; LDL-C, low-density lipoprotein cholesterol; TNF, tumor necrosis factor.

SI conversion factors: To convert hemoglobin A_1c_ to proportion of total hemoglobin, multiply by 0.01; insulin to picomoles per liter, multiply by 6.945; total cholesterol, HDL-C, and LDL-C to millimoles per liter, multiply by 0.0259; triglycerides to millimoles per liter, multiply by 0.0113; glucose to millimoles per liter, multiply by 0.0555; and C-peptide to nanomoles per liter, multiply by 0.331.

^a^ All models included a random effect for site except race and ethnicity due to lack of variability within certain sites.

^b^ Includes American Indian or Native Alaskan, Asian, and Native Hawaiian or Other Pacific Islander.

^c^ Log-transformed variables were used in the model.

### Cardiometabolic Risk Factors at Baseline

By design, individuals with MHO showed differing baseline cardiometabolic health biomarkers compared with individuals with MUO. Individuals with MHO had significantly lower concentrations of HbA_1c_, insulin, total cholesterol, LDL-C, triglycerides, glucose, and C-peptide and a lower homeostatic model assessment score and lower systolic blood pressure, as well as higher concentrations of HDL-C and adiponectin in early pregnancy (Table 1).

### Gestational Weight Gain

**Figure 2.**
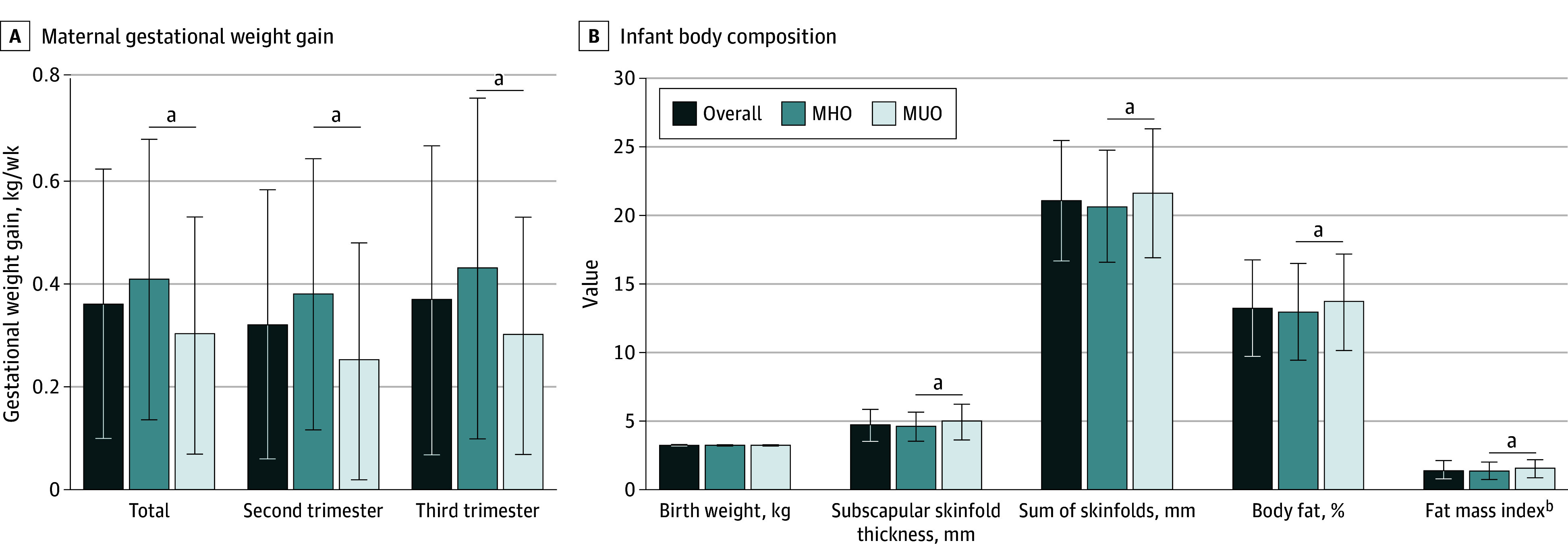
Gestational Weight Gain and Infant Body Composition Between Phenotypes in Maternal and Offspring Outcomes by Obesity Phenotype Crude or unadjusted values are presented. Error bars indicate SDs. ^a^Denotes significant differences (*P* < .05) between infants born to individuals with metabolically healthy obesity (MHO) and infants born to individuals with metabolically unhealthy obesity (MUO), after adjusting for treatment assignment, body mass index, race and ethnicity, and age at enrollment (A) and after adjusted for maternal treatment assignment, body mass index, age, race and ethnicity at maternal enrollment, gestational age at delivery, infant age, and infant sex (B). ^b^Calculated as fat mass divided by infant length squared.

Overall, individuals with MHO had significantly higher mean (SD) GWG than those with MUO (0.41 [0.27] kg/wk vs 0.30 [0.23] kg/wk; *P* < .001), a 36.7% difference (Figure 2A). Trimester-specific weight gain also differed significantly between the groups, with individuals with MHO gaining more weight than those with MUO during both the second and third trimesters. Significance remained after adjusting for treatment and BMI, race and ethnicity, and maternal age at enrollment. In the unadjusted model, more individuals with MHO exceeded IOM guidelines for weekly weight gain compared with those with MUO (68.0% [155 of 228] vs 57.0% [98 of 172]; crude: *P* = .02; and adjusted for baseline variables: *P* = .03).

### Changes to Cardiometabolic Risk Factors 

The change in maternal triglyceride, total cholesterol, glucose, insulin, HDL-C, and LDL-C levels and systolic blood pressure or diastolic blood pressure from early to late pregnancy did not differ between obesity metabolic phenotypes. At the end of pregnancy, many substrate levels remained lower in the MHO group compared with the MUO group, including total cholesterol (mean [SD], 213.5 [37.4] mg/dL vs 246.2 [54.5] mg/dL; *P* < .001) and LDL-C (mean [SD], 105.5 [30.1] mg/dL vs 139.8 [44.9] mg/dL; *P* < .001). Mean (SD) HDL-C levels remained higher among individuals with MHO than those with MUO (70.9 [17.1] mg/dL vs 54.8 [15.4]; *P* < .001). Mean (SD) triglyceride levels remained elevated but were no longer statistically significant (MHO: 186.9 [61.3] mg/dL vs MUO: 263.2 [89.5] mg/dL; *P* = .06). There were no longer differences in systolic blood pressure, glucose level, or insulin level (eFigure 2 in Supplement 1).

### Maternal and Offspring Adverse Outcomes

Individuals with MHO had a lower incidence of gestational diabetes development (9.8% [22 of 228]) compared with those with MUO (23.8% [41 of 172]) in both the crude model (*P* = .001) and after adjusting for treatment assignment, race and ethnicity, and BMI and age at enrollment (*P* = .001). There were no differences in the rates of gestational hypertension or preeclampsia, cesarean delivery, or preterm delivery (delivery prior to 32 weeks’ gestation). In addition, there were no differences in major fetal congenital malformations, fetal death, neonatal death, shoulder dystocia, or neonatal respiratory morbidity between infants born to individuals with MHO and infants born to individuals with MUO.

### Infant Outcomes

The mean (SD) gestational age at infant birth was 38.1 (4.2) weeks, and the mean (SD) age at infant study measurements was 4.1 (9.6) days of life. Overall, 46.6% of the infants enrolled (178 of 382) were male. There were no sex differences between obesity phenotypes. The mean (SD) birth weight was 3246 (488) g, and the mean (SD) length at birth was 50.1 (2.4) cm, with no differences between obesity phenotypes using the crude model and after adjusting for maternal treatment assignment, BMI, age, race and ethnicity at maternal enrollment, gestational age at delivery, infant age, and infant sex. There were no differences in the incidence of infants born large for gestational age (weight ≥90th percentile) after adjusting for maternal baseline factors, gestational age at delivery, infant age, and infant sex. The birth weight for length *z* score was lower among infants born to individuals with MHO compared with those born to individuals with MUO, though this finding was not significant (mean [SD], −0.30 [1.49] vs −0.07 [1.31]; crude: *P* = .16; and adjusted: *P* = .07).

Infants born to individuals with MHO had less adiposity. After adjusting for maternal baseline factors, gestational age at delivery, infant age, and infant sex, the mean (SD) subscapular skinfold thickness (MHO, 4.8 [1.9] mm vs MUO, 5.1 [1.2] mm; adjusted *P* = .04) and the mean (SD) sum of skinfolds (MHO, 20.7 [4.1] mm vs MUO, 21.7 [4.7] mm; adjusted *P* = .03) were lower among infants born to individuals with MHO. The mean (SD) calculated infant percentage fat (MHO, 11.7% [3.7%] vs MUO, 12.5% [3.9%]; adjusted *P* = .05), fat mass (MHO, 0.39 [0.16] kg vs MUO, 0.43 [0.18]; adjusted *P* = .04), and fat mass index (MHO, 1.53 [0.62] kg/m^2^ vs MUO, 1.67 [0.67] kg/m^2^; adjusted *P* = .04) were lower among those born to individuals with MHO (Figure 2B).

### Intervention Effects

#### Cardiometabolic Risk Differences With Intervention

There was a significant interaction effect for the changes in triglyceride levels in response to the prenatal lifestyle intervention between obesity metabolic phenotypes. Among individuals with MHO, the intervention blunted increases in triglyceride levels from early to late pregnancy, whereas a similar response was not observed among individuals with MUO. In particular, there were significant differences in the change in triglyceride levels during pregnancy in response to the prenatal lifestyle intervention between obesity phenotypes, with the MUO group assigned to control exhibiting the smallest change (mean [SE], 70.6 [7.5] mg/dL) and the MHO group assigned to control exhibiting the largest change (mean [SE], 108.3 [7.2] mg/dL) (Table 2). The intervention blunted the mean (SE) triglyceride level increases more in the MUO group than in the MHO group (81.8% [8.3%] vs 90.3% [7.4%]; *P* = .02). There were no other interactions observed in cardiometabolic risk factor changes.

**Table 2.** Metabolic Risk Factor Interactions Between Obesity Phenotype and Prenatal Interventions^a^

Risk factor	Mean (SE), percent change				*P* value for interaction
	Metabolically healthy		Metabolically unhealthy		
	Treatment	Control	Treatment		
Triglycerides	90.3 (7.4)	108.3 (7.2)	81.8 (8.3)	70.6 (7.5)	.02
Total cholesterol	29.7 (2.3)	31.2 (3.2)	23.9 (2.6)	25.3 (2.07)	.97
Glucose	0.3 (1.2)	−1.2 (1.2)	−4.8 (1.4)	−2.5 (1.2)	.10
Insulin	65.2 (9.2)	41.2 (8.8)	24.5 (10.6)	34.3 (9.3)	.05
HDL-C	3.6 (2.5)	5.7 (2.4)	−0.3 (2.7)	3.6 (2.5)	.68
LDL-C	37.1 (3.7)	36.7 (3.6)	23.7 (4.2)	25.4 (3.7)	.76
SBP	1.4 (1.3)	4.0 (1.3)	0.7 (1.5)	1.2 (1.3)	.34
DBP	3.9 (1.6)	4.9 (1.6)	3.9 (1.9)	3.0 (1.7)	.52

Abbreviations: DBP, diastolic blood pressure; HDL-C, high-density lipoprotein cholesterol; LDL-C, low-density lipoprotein cholesterol; SBP, systolic blood pressure.

^a^ All models adjusted for enrollment body mass index and age and maternal race and ethnicity. Triglycerides were measured in mg/dL; total cholesterol, in mg/dL; glucose, in mg/dL; insulin, in μIU/mL; HDL-C, in mg/dL; LDL-C, in mg/dL; SBP, in mm Hg; DBP, in mm Hg.

#### Differences in Maternal Outcomes With Intervention

**Figure 3.**
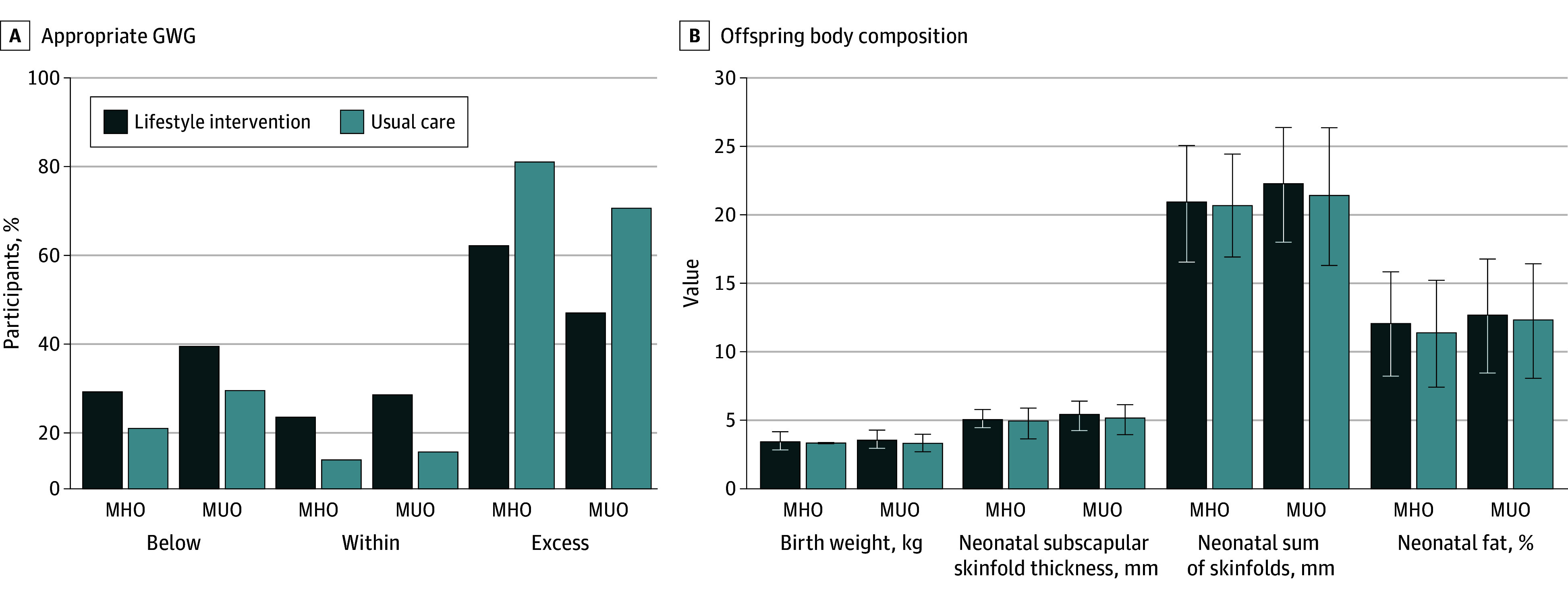
Prenatal Lifestyle Intervention and Incidence of Appropriate Gestational Weight Gain (GWG) and Offspring Body Composition Between Obesity Phenotypes Below, within, and excess denote adherence to the 2009 National Academy of Medicine gestational weight gain guidelines. MHO indicates metabolically healthy obesity; MUO, metabolically unhealthy obesity. Error bars indicate SDs.

There were no differences in total GWG, trimester-specific GWG, or incidence of appropriate GWG based on the 2009 IOM guidelines (Figure 3A) between obesity phenotypes and those enrolled in a prenatal lifestyle intervention or usual care. There were no interaction effects on the rates of gestational hypertension, preeclampsia, gestational diabetes, cesarean delivery, or preterm delivery.

#### Infant Body Composition

Infant birth weight and indicators of infant adiposity were similar within obesity phenotypes between intervention status. There was no interaction effect for weight or body composition for obesity phenotype and intervention status (Figure 3B). In addition, there were no interaction effects on major fetal congenital malformations, fetal death, neonatal death, shoulder dystocia, or neonatal respiratory morbidity between infants born to individuals with MHO or those born to individuals with MUO.

## Discussion

In this analysis of the randomized clinical trial of a prenatal IBT on GWG, we identified different obesity metabolic phenotypes among pregnant individuals. These data validated the existence of differential metabolic responses throughout pregnancy on the basis of the early pregnancy metabolic milieu. We observed that with respect to weight changes across pregnancy, individuals with MUO had a 36.7% lower GWG compared with individuals with MHO, regardless of intervention. We hypothesized that downstream maternal, birth, and offspring outcomes would be influenced by early pregnancy maternal metabolic phenotypes. Individuals with MUO had a higher rate of gestational diabetes and infants born with significantly more body fat compared with infants born to individuals with MHO. This finding was evidenced by a higher body fat percentage, body fat mass, and fat mass index among infants born to individuals with MUO. With regard to the differential response to a prenatal IBT lifestyle intervention, we rejected our primary hypothesis, which proposed that individuals with MUO would account for the lack of robust outcomes in response to prenatal lifestyle interventions. Results showed similar maternal adverse outcomes between the MUO and MHO groups in response to a prenatal lifestyle intervention.

These data provide insights into how prenatal lifestyle interventions affect different pregnant individuals with varying baseline metabolic health. First, infants born to individuals with an a metabolically unhealthy obesity phenotype in early pregnancy are born similar in size but with greater adiposity compared with those born to individuals with a metabolically healthy obesity phenotype in early pregnancy. There was also indication of increased central adiposity among infants born to individuals with an unhealthy early pregnancy phenotype, as evidenced by greater measurements of subscapular thickness.^21^ These outcomes occurred independent of early pregnancy weight or amount of GWG. The presence of worsened maternal outcomes (eg, higher incidence of gestational diabetes) and infant adiposity among those with MUO, despite lower rates of GWG, is unexpected. Higher rates of GWG have been repeatedly associated with such adverse outcomes, yet the present association does not support these established findings. It is likely that metabolic health that typically underlies excess weight gain, rather than weight gain itself, is the primary driver of such adverse outcomes. Individuals with MUO present with elevated glucose and lipid levels—the 2 primary sources of fetal nutrition—across the entire duration of pregnancy. It is likely that prolonged fetal exposure to these growth-promoting substrates contributes to early epigenetic programming and adipocyte development in the fetus. Cellular models using umbilical cord mesenchymal stem cells support this hypothesis, showing that cells cultured from those with obesity plus maternal metabolic inefficiency have the greatest impairments in fetal fat oxidation.^7^ Perturbations to metabolic systems during pregnancy include elevations in fasting glucose and triglyceride levels and insulin resistance.^22^ It has been suggested that the typical prenatal intervention intensity may be too low to result in clinically significant differences in weight gain, thus failing to produce meaningful clinical outcomes in the mother and infant.^27^ Although the USPSTF criteria support that the current trials are considered high intensity, the intervention was not initiated until the start of the second trimester. Thus, the combination of intensity concomitant with late initiation may have failed to influence the pregnancy metabolic milieu. As a result, the excess metabolic load of pregnancy in addition to the underlying metabolically stressed milieu was shown to be detrimental to the development of pregnancy complications and optimal fetal growth.

Current health recommendations support IBT interventions that counsel weight management during pregnancy. Recent USPSTF recommendations propose that clinicians offer behavioral counseling aimed at preventing excess GWG across at least 12 intervention sessions during pregnancy.^23^ Although excess GWG is associated with worsened maternal and offspring outcomes, interventions successful at reducing weight gain have repeatedly failed to produce meaningful clinical outcomes. Although each trial in the presented consortium of trials was unique, they were similar in their approach to recommend diet and physical activity modifications to achieve appropriate GWG. Therefore, with the current analysis, it is not possible to identify any unique strategies that would achieve downstream outcomes. However, these data provide strong evidence that lifestyle interventions should instead be focused on precision medicine strategies, which, in addition to supporting appropriate GWG, have the potential to alter the maternal metabolic milieu in pregnancy. Such strategies may include specific diet modifications, exercise recommendations, and altering the eating window—known powerful modulators of the metabolic milieu in nonpregnant adults.^24,25^

### Strengths and Limitations

This study has some strengths. A major strength is the large, diverse sample with richness of metabolic parameters collected early and late in pregnancy. Furthermore, the statistical division of 2 phenotypes that excludes those with obesity plus 1 underlying risk factor created 2 dichotomous groups for study.

Several limitations also need to be considered. The trial excluded individuals with diabetes or prediabetes (elevated HbA_1c_ level) and hypertension, thus limiting most of our participants to those with mild elevations in HbA_1c_ level and hyperlipidemia (eFigure 1B in Supplement 2). Therefore, with the present study design, we did not observe individuals with extreme metabolic complications and likely attenuated the number of participants in the MUO group.

## Conclusions

This preplanned secondary analysis of a randomized clinical trial found that prenatal lifestyle interventions appeared equally effective at influencing GWG across 2 identified metabolic phenotypes and with no differences on maternal and delivery outcomes. Offspring health, as evidenced by higher adiposity, was worse in the group that gained less weight during pregnancy. Gestational weight gain may be less important than the maternal metabolic milieu in estimating perinatal outcomes and infant health, highlighting the need for prenatal interventions that focus on optimizing maternal metabolic health

## Notes

Supplement 1.
Trial Protocol and Statistical Analysis Plan

Supplement 2. **eTable 1. **Interventions Included in the LIFE-Moms Consortium Trial to Reduce Gestational Weight Gain **eTable 2. **Attainment to the 2009 NAM GWG by Obesity Phenotype and Randomized Group **eTable 3. **Infant Outcomes by Obesity Phenotype and Randomized Group **eFigure 1.** Criteria for Metabolic Health Phenotype Classification **eFigure 2.** Changes in Maternal Substrates and Blood Pressures Through Pregnancy

Supplement 3.
Data Sharing Statement
