# Supplementary material for: Metabolic Health and Heterogenous Outcomes of Prenatal Interventions: A Secondary Analysis of a Randomized Clinical Trial
Source: JAMA Netw Open. 2025 Aug 21;8(8):e2528264. doi: 10.1001/jamanetworkopen.2025.28264 (PMC12371516; doi:10.1001/jamanetworkopen.2025.28264)
Supplement: Supplement 1. — Trial Protocol and Statistical Analysis Plan [file jamanetwopen-e2528264-s001.pdf]

JNO25-5272: Supplementary Materials

This supplement contains the following:

1. Original consortium protocol
2. Statistical analysis plan for the present investigator-initiated analysis

Lifestyle Interventions for Expectant Moms
LIFE-Moms

Protocol

National Institutes of Health

Prepared by the

Research Coordinating Unit

The Biostatistics Center
The George Washington University
6110 Executive Boulevard, Suite 750
Rockville, MD 20852
(301) 881-9260

May 23, 2012

Table of Contents

Protocol **1**
1 Introduction **2**
1.1 Abstract 2
1.2 Purpose of the Overview Protocol 2
2 Consortium Objective **3**
3 Clinical Centers and Collaborating Units **4**
3.1 Clinical Centers 4
3.2 RCU 5
3.3 NIH 5
4 Committees **6**
4.1 Steering Committee 6
4.2 Standing Committees 6
4.3 Subcommittees 7
4.4 Core/Shared Measures 8
5 Study Design **9**
5.1 Primary Research Question 9
5.2 Design Summary 9
5.3 Study Population and Eligibility Criteria 9
5.3.1 Source Population 9
5.3.2 Core Inclusion Criteria 10
5.3.3 Gestational Age Determination 10
5.3.4 Core Exclusion Criteria 11
5.4 Informed Consent Procedures 12
5.5 Randomization Method and Masking 12
6 Study Procedures **13**
6.1 Screening/Recruitment 13
6.2 Randomization 13
6.3 Baseline Procedures 13
6.4 Study Procedures 15
6.4.1 Condition 15
6.4.2 Standardization of Intervention Delivery 15
6.4.3 Procedures & Visit Schedule 16
6.5 Patient Management and Follow-up 18
6.6 Safety Monitoring / Adverse Event Reporting 18
6.6.1 Definitions of AE/SAE 20
6.6.2 Rules for Intervention Termination / Modification 20
6.7 Study Outcome Measures and Ascertainment 21
6.7.1 Primary Outcome 21
6.7.2 Maternal Secondary Outcomes 21
6.7.3 Fetal and Neonatal Secondary Outcomes 21
6.7.4 Infant Secondary Outcomes 21
7 Statistical Considerations **22**
7.1 Data Relevant to the Primary Outcome .... **Error! Bookmark not defined.**
7.2 Sample Size and Power .... **Error! Bookmark not defined.**
7.3 Interim Monitoring Plan for the Sites .... **Error! Bookmark not defined.**
7.4 Analysis Plan 22
7.4.1 Methodology 22
7.4.2 Racial/Ethnic Subgroup Analysis .... **Error! Bookmark not defined.**

7.4.3 Handling Missing or Incomplete Data ... 22
8 Data Collection ... 23
8.1 Data Collection Forms ... 23
8.2 Data Entry and Management System ... 23
8.2.1 Quality Control ... 23
8.2.2 Confidentiality and Data Security ... 24
8.2.3 Sharing of Data for Common Measures ... 25
8.2.4 Transfer of Center-Specific Data to NIDDK Repository ... 25
8.3 Performance Monitoring ... 25
9 Study Timetable ... 26
9.1 Training and Certification ... 26
9.2 Recruitment and Data Collection Period ... 26
9.3 Final Analysis ... 26
Appendix A. Intervention by Component ... 27
References ... 33

May 23, 2012

Preface

This overview of the LIFE-Moms Consortium describes the design and organization of the overall effort. This overview will be maintained by the LIFE-Moms Research Coordinating Unit (RCU) at The George Washington University throughout the course of the project through new releases of the entire overview, or issuance of updates, or in the form of supplemental memoranda.

1 Introduction

1.1 Abstract

The overall goal of the Lifestyle Interventions in Overweight and Obese Pregnant Women Consortium is to identify effective behavioral and lifestyle interventions that will improve weight, glycemic control and other-pregnancy-related outcomes in obese and overweight pregnant women, and determine whether these interventions reduce obesity and metabolic abnormalities in their children.

There are seven studies which are part of the Lifestyle Interventions in Overweight and Obese Pregnant Women Consortium, known as Lifestyle Interventions for Expectant Moms or ‘LIFE-Moms’. The Consortium is funded by the National Institute of Diabetes and Digestive and Kidney Diseases (NIDDK), the National Heart, Lung, and Blood Institute (NHLBI), the *Eunice Kennedy Shriver* National Institute of Child Health and Human Development (NICHD), and the National Center for Complementary and Alternative Medicine (NCCAM), with additional support from the Office of Research on Women’s Health (ORWH) and Office of Behavioral and Social Science Research (OBSSR).

1.2 Purpose of the Overview Protocol

The LIFE-Moms Consortium will work collaboratively to ensure the clinical and scientific integrity and success of this project. The LIFE-Moms Consortium conduct studies testing behavioral/lifestyle interventions in overweight and obese pregnant women designed to improve weight and metabolic outcomes in both the pregnant women and their offspring. The Overview Protocol serves to document the infrastructure by which the collaborative development of protocols, data acquisition, standardization and sharing of data and specimens will occur, including the planned contribution to data and specimen repositories.

2 Consortium Objective

The Consortium is a collaboration between the seven clinical centers, the Research Coordinating Unit and the funding Institutes and Centers of NIH. Funding is awarded to each clinical center and the RCU under the cooperative agreement mechanism, thus there is substantial NIH programmatic involvement in all aspects of the Consortium.

Although each clinical center is conducting a separate trial, the goal of the collaboration is to maximize the value of the individual trials by identifying core measures to be collected across all studies; ensuring consistency of procedures, definitions and data collection; harmonizing design elements of the trials and the proposed interventions; and jointly monitoring progress and solving problems as the trials progress. This will facilitate the ability to combine the data and resources to different approaches when there is not enough evidence to test one single approach in a multi-center trial. The organization of the group is built around these goals.

3 Clinical Centers and Collaborating Units

3.1 Clinical Centers

The LIFE-Moms Consortium consists of seven clinical centers which are listed below.

**Center 1**	**California Polytechnic Institute State University & Brown University (CP/B)**
Site Protocol:	Healthy Beginnings
Principal Investigators:	Suzanne Phelan (Cal Poly)<br>Rena R. Wing (Brown)

**Center 2**	**St. Luke's – Roosevelt (St. Lukes)**
Site Protocol:	Lifestyle Interventions For Two (LIFT)
Principal Investigators:	Xavier Pi-Sunyer<br>Dympna Gallagher

**Center 3**	**University of Puerto Rico (UPR)**
Site Protocol:	Pregnancy and EARly Lifestyle improvement Study (PEARLS)
Principal Investigators:	Kaumudi Joshipura<br>Frank Hu

**Center 4**	**Northwestern University (NW)**
Site Protocol:	Maternal-Offspring Metabolics: Family Intervention Trial (MOMFIT)
Principal Investigators:	Linda Van Horn<br>Alan Peaceman

**Center 5**	**Washington University in St. Louis (WU)**
Site Protocol:	Weight Management in Obese Pregnant Underserved African American Women
Principal Investigators:	Sam Klein<br>Debra Haire-Joshu<br>Kelle Moley

**Center 6**	**Pennington Biomedical Research Center (PBRC)**
Site Protocol:	Expecting Success: Personalized Management of Body Weight during Pregnancy
Principal Investigators:	Leanne Redman<br>Corby Martin

**Center 7**	**NIDDK-Phoenix Indian Medical Center (PIMC)**
Site Protocol:	Lifestyle Interventions for Expectant Moms (LIFE - Moms)-Phoenix
Principal Investigator:	William Knowler

3.2 RCU

RCU	Research Coordinating Unit
George Washington University Biostatistics Center	
Principal Investigators:	Elizabeth Thom<br>Rebecca Clifton

3.3 NIH

NIH	National Institutes of Health
NIDDK:	Consortium Program Scientist: Mary Evans<br>Consortium Program Official: Mary Horlick<br>Additional Project Scientists: Barbara Linder, Sue Yanovski<br>Sites primarily funded by NIDDK: St. Luke's – Roosevelt, Washington University in St. Louis, Pennington Biomedical Research Center, NIDDK-Phoenix Indian Medical Center
NICHD:	Program Official: Caroline Signore<br>Site primarily funded by NICHD: University of Puerto Rico
NHLBI:	Program Official: Sonia Arteaga<br>Site primarily funded by NHLBI: California Polytechnic Institute State University & Brown University, Northwestern University

4 Committees

4.1 Steering Committee

The Steering Committee is the governing body for the Consortium and is composed of the Principal Investigator(s) of each clinical center, the RCU, and the NIH. The Steering Committee has primary responsibility to design research activities, establish priorities, develop common protocols and manuals, questionnaires and other data recording forms, establish and maintain quality control among awardees, review progress, monitor patient accrual, coordinate and standardize data management, and cooperate on the publication of results. Major scientific decisions regarding the core data will be determined by the Steering Committee

Two co-Chairs have been appointed by NIH from among the members of the Steering Committee. Each clinical center, the RCU, and the NIH has one vote. In general, the Steering Committee will meet in-person twice each year, and more frequently by teleconference.

Executive Committee. This subgroup of the Steering Committee consists of the NIH Program Scientist and two Program Officials, the RCU Principal Investigator(s), and the co-Chairs of the Steering Committee. This committee meets more frequently than the Steering Committee. The purpose of the Executive Committee is to discuss emerging issues between Steering Committee meetings and to recommend solutions and policies to be put before the Steering Committee for discussion and vote.

4.2 Standing Committees

Four standing committees have been established as follows:

Recruitment & Retention Committee. This committee will develop plans to track recruitment and retention across all clinical sites. Other activities may include: developing recruitment and retention tracking tools, creating a recruitment and retention report, providing a mechanism to share the best practices and lessons learned about recruitment strategies, and monitoring/trouble-shooting study-specific recruitment issues.

Ancillary Studies Committee This committee is charged with developing a policy for submission/evaluation of ancillary proposals for external funding to use core study data and/or biospecimens. The committee will review ancillary study proposals and make recommendations to the Steering Committee.

Publications & Presentations Committee. This group will develop a policy for publications and presentations specific to core measures and analyses and oversee all publications and presentations that emanate from the Consortium as a whole. The Committee is also charged with identifying opportunities for other Consortium-wide publications

Safety Committee. The Safety Committee includes expertise in relevant disciplines, including obstetrics and pediatrics. This committee is charged with establishing common definitions for safety-related events and methods for ascertaining and reporting such events as required by local IRBs and the funding institutes. The committee is charged with recommending definitions of safety-related exclusion criteria, adverse events and serious adverse events, alert values for participant assessments, and tracking medication use. The Safety Committee will make recommendations to the Steering Committee and develop training procedures as appropriate. The Safety Committee will also review all adverse events reported on a regular basis. Data will be tabulated by the RCU for distribution to the committee.

4.3 Subcommittees

Subcommittees and working groups of the subcommittees are formed as needed to determine core and shared measures and procedures, and how data and resources derived from core measures and procedures will be processed, analyzed or used. Subcommittees may become inactive once their goals have been accomplished.

At a minimum, a representative from each clinical center, NIH and the RCU will sit on each subcommittee. Every PI is expected to serve on at least one subcommittee (or standing committee), but clinic investigators/staff are also eligible. Individuals may serve on more than one committee.

Eight subcommittees have been established as follows:

The **Physical and Clinical Measures Committee** will identify and provide recommendations for common measures that will be collected or assessed in all participants (separate lists for mothers and offspring). The committee will consider data usually captured as part of standard of care, as well as research measures. The committee will confirm that all measures are collected and reported similarly across sites. The committee will also develop standardized protocols and training procedures for the recommended measures, as necessary.

The **Survey/Questionnaires Committee** will evaluate and propose common questions/questionnaires and survey instruments to capture data of interest to the entire consortium. Examples include demographic characteristics, weight history, behavioral measures (mindfulness, etc.), quality of life, depression, and sleep quality.

The **Diet & Physical Activity Assessment Committee** will be composed of experts in dietary and physical activity assessment. This committee will discuss and provide recommendations for uniform methodologies that could be used to evaluate dietary intake and physical activity across all of the studies. The committee will also consider feasibility (ppt/staff time and burden) and cost associated with these methodologies.

The **Intervention Committee** will discuss the similarities and differences among the interventions, and the translation and sustainability of the interventions. The committee will also evaluate common cost measures and uptake within the community.

The **Design, Data Quality and Analysis Committee** will draw together experts in clinical trial design, data management and data analysis. It aims to facilitate data sharing, pooled data analyses, and potential meta-analyses among trials by identifying common approaches to trial design, data management and quality, and statistical analysis. Topics the committee will consider include evaluating current data quality measurements and develop new data quality measurements (as necessary), establishing data quality reports that will be part of the DSMB template, developing strategies that facilitate data sharing across trials, recommending approaches to primary data analysis that will be common across the trials, developing standardized methods to handle missing and censored data that will be shared across all trials, and responding to analytic issues raised by the DSMB.

The **Biospecimen Committee** will consider research questions that the consortium is positioned to address with serial specimens from participating women and their offspring. It aims to establish a standard list and schedule of specimens to be obtained, taking feasibility and study timelines into account. The committee will make specific recommendations for specimen collection, preparation, and storage recognizing that analyses of the specimens may not be possible with current funding, but that a well-planned bio-repository linked to study wide pregnancy, postpartum, and early life data is a valuable resource for further investigation.

The **Data Management Committee** will review each site's data management system and discuss important issues regarding data entry using the RCU's web-based data management system (MIDAS) or by file upload via secure FTP.

A **Coordinators Committee** will be formed to discuss the manual of procedures for the Core measures and recruitment strategies.

4.4 Core/Shared Measures

Each of the following subcommittees met 4-5 times in person or by teleconference to propose Core measures/specimens and inclusion/exclusion criteria: Physical & Clinical Measures, Survey/Questionnaires, Diet & Physical Assessment, Design Data Quality & Analysis, and Biospecimens committees. Key measures and procedures that will be common to all of the trials are designated as 'Core' and will provide the basis for Consortium wide evaluations. 'Shared' was described as measures and procedures which 2 or more sites were collecting. The LIFE-Moms Steering Committee reviewed and approved the final list of Core measures. The Consortium also standardized measures and procedures for a limited number of Shared measures.

5 Study Design

5.1 Primary Research Question

Can prenatal diet, behavioral, and lifestyle interventions among overweight/obese (OW/OB) pregnant women reduce the incidence of excess gestational weight gain above the 2009 Institute of Medicine gestational weight gain guidelines?

For the purpose of this primary outcome, gestational weight gain is defined as the difference between the maternal weight measured at the 36 week visit and the baseline weight.

5.2 Design Summary

Seven different randomized clinical trials (RCT) plan to enroll 2,086 ethnically diverse OW/OB pregnant women over 18 years of age. The interventions vary among the seven different sites. A description of each intervention may be found in Appendix A.

5.3 Study Population and Eligibility Criteria

The sample sizes for each of the seven studies are the following:

Site	Sample Size
CP/B	350
St. Lukes	210
UPR	400
NW	300
WU	266
PBRC	306
NIDDK-PIMC	200
Total	2032

5.3.1 Source Population

All seven sites are targeting minorities and/or participants from low income backgrounds.

Target Population	Overall	CP/B	St. Lukes	UPR	NW	WU	PBRC	PIMC
%White	33%	50%	55%		63%		56%	
%African American	21%		20%		12%	100%	37%	
%Latino	34%	50%	15%	100%	18%		3%	
%Asian/Pacific Islander	1%		8%				4%	
%American Indians	10%							100%
%Other	1%		2%		7%			

Site	Deliveries per year
CP/B	4,600
St. Lukes	6,500
UPR	1,000
NW	12,000
WU	4,000
PBRC	8,000
NIDDK-PIMC	700
Total	36,800

5.3.2 Core Inclusion Criteria

Below are the Core inclusion criteria for the Consortium. Additional inclusion criteria may be added by each site.

- Singleton viable pregnancy. A twin pregnancy reduced to singleton before 14^0 weeks by project gestational age (see Section 3.4.3 below) is acceptable. An ultrasound must be conducted before randomization that shows a fetal heartbeat; there should be no evidence of more than one fetus on the most recent pre-randomization ultrasound. *
- Gestational age at randomization no earlier than 9 weeks 0 days and no later than 15 weeks 6 days based on an algorithm (see Section 3.4.3 below) that compares the LMP date and data from the earliest ultrasound*
- Body mass index based on first trimester measured weight and on measured height. The earliest weight measurement before randomization, measured specifically for the study will be used. Reported pre-pregnancy weight will not be used to determine eligibility because of the potential for inaccuracy. If the earliest weight measurement is conducted at 14^0 to 14^6 weeks or 15^0 to 15^6 weeks, 1lb or 2lb will be subtracted from the measured weight respectively, to adjust to a first trimester weight.*

Site	BMI Inclusion Criteria (kg/m2)
CP/B	≥ 25
St. Lukes	≥25 - ≤ 35
UPR	≥ 25
NW	≥25 - < 35
WU	≥30 - < 45
PBRC	≥25 - < 40
NIDDK-PIMC	≥25

- Age cutoffs have been included in the inclusion criteria or exclusion criteria depending on the site*

Site	Age Inclusion Criteria
CP/B	≥ 18
St. Lukes	18-40
UPR	≥ 18
NW	18-45
WU	18-35
PBRC	18-40
NIDDK-PIMC	≥ 18

5.3.3 Gestational Age Determination

Gestational age is determined as follows, and is denoted “project gestational age”. The “project EDC”, which is based on the project gestational age, cannot be revised once a determination has been made.

* Core eligibility criterion for the LIFE-Moms Consortium

1. The first day of the last menstrual period (LMP) is determined, and a judgment made as to whether or not the patient has a "sure" LMP date.
2. If the LMP date is unsure, the ultrasound measurements obtained at the patient's first dating ultrasound examination are used to determine the project gestational age, by the standard method of ultrasound gestational age determination at that institution.
3. If the LMP date is sure, project gestational age is determined by a comparison between the gestational age by LMP and by the earliest dating ultrasound.
4. If the earliest dating ultrasound confirms the gestational age by LMP within ± 7 days
   - The LMP-derived gestational age is used to determine the project gestational age.
   - If the ultrasound determined gestational age does not confirm the LMP generated gestational age within ± 7 days, the ultrasound is used to determine the project gestational age.

In the case of in-vitro fertilization, project gestational age is calculated as days elapsed since embryo transfer plus embryo age at transfer. Project EDC is defined as date of embryo transfer –age of embryo at transfer +266 days.

5.3.4 Core Exclusion Criteria

Below are the Core exclusion criteria for the Consortium. Additional exclusion criteria may be added by each site.

- Age less than 18 years because of the potential for growth during pregnancy*
- Diagnosis of diabetes prior to pregnancy, or an Hb A1c ≥ 6.5 % or other test result < if glucose tolerance test being conducted before randomization insert study specific criteria here> suggestive of pre-pregnancy diabetes. All potential participants will have an Hb A1c performed prior to randomization.*
- Known fetal anomaly *
- Planned termination of pregnancy*
- History of three or more consecutive first trimester miscarriages*
- Past history of anorexia or bulimia by medical history or patient report. Binge eating disorder (BED) is not an exclusion.*
- Current eating disorder diagnosed by EDE-Q questions 2-4 and confirmed after discussion with the participant by study staff*
- Actively suicidal defined as a value ≥ 2 on the BDI-II question 9*
- Prior or planned (within 1 year of expected delivery) bariatric surgery*
- Current use of one or more of the following medications: *
  - Metformin
  - Systemic steroids
  - Antipsychotic agents (e.g., Abilify, Haldol, Risperdal, Seroquel, Zyprexa)
  - Anti-seizure medications or mood stabilizers that would be expected to have a significant impact on body weight (e.g., Depakote, Lamictal, Lithium, Neurontin, Tegretol, Topamax, Keppra)
  - Medications for ADHD including amphetamines and methylphenidate
- Continued use of weight loss medication including OTC and dietary supplements for weight loss (e.g., Adipex, Suprenza, Tenuate, Xenical, Alli, conjugated linoleic acid, Hoodia, Green tea extract, Guar gum, HydroxyCut, Sensa, Corti-slim, Chromium, chitosan, Bitter orange) *
- Contraindications to aerobic exercise in pregnancy specified in the ACOG Committee Opinion #267, 2002 (reaffirmed 2009) *

  - Hemodynamically significant heart disease defined as an AHA class II (short of breath with exercise) or greater
  - Restrictive lung disease (e.g. pulmonary fibrosis)
  - Poorly controlled hyperthyroidism
  - Poorly controlled seizure disorder
  - Poorly controlled hypertension defined as a blood pressure $\geq$ 160/110
  - History of extreme sedentary lifestyle (e.g. bed bound)
  - Orthopedic limitations to aerobic exercise
  - Severe anemia defined as a hemoglobin < 8
- Participation in another interventional study that influences weight control*
- Enrollment in this trial in a previous pregnancy*
- Intention of the participant or of the care provider for the delivery to be outside the LIFE-Moms Consortium hospital*
- Participant's unwillingness or inability to commit to a 1 year follow-up of herself or her child, including planning to move away.*

Some sites have chosen to add additional exclusion criteria (e.g. smoking, nulliparous) or have more restrictive exclusion criteria (e.g. exclude binge eating disorder (BED)).

5.4 Informed Consent Procedures

Information about each study's Informed Consent can be found in the specific protocol. The RCU will collect each center's final Informed Consent forms along with IRB approvals.

5.5 Randomization Method and Masking

Site	Randomization Method	Stratification	Masking
CP/B	Variable blocks	Site, Ethnicity/Race	Research assistants blinded during assessment procedures
St. Lukes	Simple randomization Random number generator; <0.5-A, ≥0.5-B	None	Data managers; some data collection procedures collected by blinded staff
UPR	Variable blocks	None	Staff conducing assessments will be blinded
NW	Variable blocks	None	
WU	Fixed blocks	None	Outcomes personnel will be masked
PBRC	Variable blocks	Overweight / Obese	Study coordinator; staff members who perform clinical testing will be blinded
NIDDK-PIMC	Variable blocks	Nondiabetic / gestational diabetic based on OGTT	Laboratory and other outcomes staff will be blinded

6 Study Procedures

6.1 Screening/Recruitment

Research staff will review the inclusion/exclusion criteria against each potential participant's medical and obstetrical history. If no dating ultrasound with evidence of a fetal heartbeat has previously been conducted, one must be conducted and gestational age criteria confirmed before a participant can be randomized.

Weight will be measured without shoes, wearing light clothing on a calibrated beam balance scale and height will be measured with a stadiometer at the baseline visit according to a standardized protocol to confirm the BMI eligibility criteria.

If the potential participant has not had a HbA1c test during the current pregnancy, blood will be collected and tested to ensure eligibility. The patient will be asked questions from the Eating Disorders Examination-Questionnaire[1] (EDE-Q) and the Beck Depression Inventory II[2] (BDI-II) will be administered to confirm eligibility in the trial as well as to assess depression.

Additional screening and recruitment procedures are site specific.

6.2 Randomization

Site	Randomization Process
CP/B	Sealed envelopes
St. Lukes	Phone call
UPR	Sealed envelopes
NW	Computer program
WU	Sealed envelopes
PBRC	Sealed envelopes
NIDDK-PIMC	Sealed envelopes

6.3 Baseline Procedures

In addition to information collected for eligibility, the following Core data will be obtained by participant and chart review:

- Demographic information: maternal date of birth, race and ethnicity, marital status, family size, household type, income, occupational status, TV, telephone and computer ownership, internet/computer access, cell phone usage and technology, maternity leave, education/literacy.
- Paternal demographic information: age at maternal enrollment, race, ethnicity, body type as identified by participant using standard silhouettes
- Medical history: self-reported pre-pregnancy weight, underlying medical conditions, current medications
- Obstetrical history: to include gravidity, parity, and a description of previous births (year, birth weight, gestational age at delivery, weight gain, gestational diabetes, preeclampsia, birth trauma. A Modified-Pregnancy-Unique Quantification of Emesis and Nausea Index[3] (PUQE) questionnaire, which assesses the severity of nausea and vomiting of pregnancy, will be given at baseline.
- Social history: alcohol use, tobacco use

Blood Pressure (BP). Seated BP will be measured after a 5 minute rest according to a standardized protocol.

Maternal physical activity. The *ActiGraph GT3X+* accelerometer will be used to measure maternal activity and sleep objectively during gestation for no less than seven days. The monitor records time varying accelerations ranging in magnitude from +/- 6 g's. The accelerometer output is sampled by a twelve-bit Analog to Digital Convertor and the raw acceleration is then stored in flash memory for future analysis. The GT3X+ is small and lightweight (19g) and can be worn at the hip, wrist, or ankle. The LIFE-Moms consortium will use wrist-placement to minimize participant inconvenience and to better measure sleep.

Questionnaires

- *Household food security* will be measured with the first two questions of the USDA Food Security Module subscale
- *Sleep:* A sleep questionnaire, including questions regarding sleep schedule, quantity, quality, habits and disorders and sleepiness
- *Frequency of weighing.* Participants will be asked to indicate how frequently they had weighed themselves during the past month using a 7-point scale ranging from *several times a day* to *never*[4-5]
- *Maternal Sedentary Behavior* will be measured with Item 39 from the Nurse's Health Study II Questionnaire (http://www.channing.harvard.edu/nhs/questionnaires/pdfs/NHSII/2009.pdf). This item includes 5 questions that ask respondents to rate the average hours per week over the past year that they engaged in certain sedentary behaviors.
- *Maternal Physical Activity Behavior* (SHARED) will be measured with Items 37, 38, and 40 from the Nurse's Health Study II Questionnaire (http://www.channing.harvard.edu/nhs/questionnaires/pdfs/NHSII/2009.pdf). Item 37 asks participants to identify usual walking pace from 5 speed/perceived intensity options. Item 38 asks participants to identify time spent engaging in 10 different activities (walking, jogging, running, bicycling, racquet sports, lap swimming, aerobic dance, low intensity activities like yoga, other vigorous activities, and resistance training). Item 40 asks how many days per week the participant engages in exercise. The instructions for these items will be changed to the following, which allows participants to rate their physical activity behavior over the past year or since the last time that they completed this questionnaire: "DURING THE PAST YEAR OR SINCE THE LAST TIME YOU COMPLETED THIS QUESTIONNAIRE…"
- *Health Survey (SF-12).* The SF-12[6] (SHARED) is a 12-item self-report measure of quality of life. It was derived from the original 36-item measure, and taps a variety of domains including physical functioning, bodily pain, general health, vitality, social functioning, physical and emotional functioning, and mental health functioning. The SF-12 also provides two composite indices of physical health and mental health.
- *Mindful Eating Questionnaire (MEQ).* (SHARED) The construct of mindfulness refers to a nonjudgmental awareness of physical and emotional sensations associated with eating that, if recognized may help a person identify and respond appropriately to satiety[7-10]. A 28 item scale was developed and validated by Framson, et al[11] as an initial step towards characterizing and measuring mindful eating. It addresses disinhibition, awareness, external cues, emotional responses and distraction but is distinct from cognitive restraint. LIFE Moms can assess and differentiate over time whether the role of mindful eating plays a role in facilitating adherence to an energy balanced diet.

- *Built Environment in home neighborhood*. (SHARED)The Physical Activity Neighborhood Environment Survey (PANES) is a well-validated questionnaire that assesses environmental factors for walking and bicycling in your neighborhoods[12].
- *Automated Self-Administered 24-Hour Dietary Recall (ASA24). (SHARED)* The ASA24 Recall[13] will be used to query maternal nutritional status, dietary change, and dietary supplement intake. The web based dietary interview incorporates the automated multiple pass method by the US Department of Agriculture (USDA) and food portion photography. The software is linked to the updates of multiple databases including the Food and Nutrient Database for Dietary Studies (FNNDS 4.1). Interviews will take approximately 30-45 minutes to administer. Recommended collection points for prenatal are baseline, 35±3weeks, and 1 year postnatal.
- *The Eating Behaviors questionnaire* (SHARED) assesses a) consumption of sugar sweetened beverages (SSB), b) meals consumed away from home, and c) daily meal patterns. Six questions assess type and frequency of SSB. Three questions assess how often meals are eaten at fast food restaurants, full service restaurants, and all you can eat buffets. One question assess how often meals are consumed that are prepared in the home. Seven questions assess daily meal patterns by asking the frequency of consumption of specific meals (e.g., breakfast, evening snack) during a typical week. The Eating Behaviors questionnaire was assembled by the EARLY* investigators and includes questions from other dietary assessments (DHQ-II) and questions developed by the EARLY investigators.

  *Early Adult Reduction of weight through Lifestyle interventions trials (EARLY) is a consortium of 7 trials targeting weight control among young adults aged 18-35.

Maternal blood and urine. Samples will be collected in the morning following an overnight fast. A total of thirty-two (32) mL of blood will be collected with approximately 15 mL used to obtain archived samples of serum and plasma, 8 mL for DNA extraction, 3 mL for RNA extraction and 5 mL to measure a metabolic panel comprised of glucose, insulin, lipid panel and C-reactive protein. A total of five (5) mL of urine will be collected. All samples will be stored at the NIDDK Repository.

Additional baseline procedures and questionnaires will be site specific.

6.4 Study Procedures

6.4.1 Condition

Description of each site's intervention and management of the control groups are described in each study's protocol and summarized in Appendix A.

6.4.2 Standardization of Intervention Delivery

Each intervention will be delivered by trained study staff.

6.4.3 Procedures & Visit Schedule

Core study visits are baseline (described above), 24,0-27,6 weeks of gestation, 35,0-36,6 weeks, neonatal period and 48-56 weeks postpartum.

	Measurement	24,0-27,6 wks	35,0-36,6 wks	Neonatal Period	48-56 wks postpartum
Maternal medical information and demographics	Current medications		X		X
	Demographics				X (if changed)
Maternal Food Intake	ASA-24				X
Maternal Anthropomorphics[1]	Weight	X	X[1]		X
	Blood Pressure	X	X		X
	BODPOD				X
Maternal Metabolic Testing	75gm 2-hr Oral Glucose Tolerance Test	X			
	Blood for archive, DNA, RNA, Metabolic panel		X		X
	Spot urine for Ketone testing	X	X		
Maternal Activity	Actigraph		X		
Questionnaires	Household food security				X
	Sleep		X		X
	Health Survey (SF12)				X
	Mindful Eating (MEQ)		X		X
	PANES				X
	Eating Behaviors				X
	Frequency of weighing	X	X		X
	Maternal Sedentary Behavior	X	X		X
	Maternal PA Items		X		X
	Infant Milestones				X
Breastfeeding / Infant Eating Styles	Breastfeeding initiation and continuation				X
	Feeding practices				X
	Long FFQ				X
Infant Anthropomorphics	Length/Height/ Weight/Skin fold			24 hrs – 1 week of life	X
	PEAPOD				X
	Blood pressure				X
Infant Demographics	Race / Ethnicity			X	
	Child TV Viewing				X

[1] Antenatal records will be reviewed for the closest prenatal weight to 35 weeks measured at the maternity care visit if the 35,0-36,6 study visit is missed.

Ketones/ Glucose tolerance Maternal urine will be tested for the presence of ketones at 24,0-27,6 weeks, and 35,0-36,6 weeks. Glucose tolerance will be measured by a 75 gm 2 hour Oral Glucose Tolerance Test at 24,0-27,6 weeks.

Pregnancy complication, delivery and neonatal data will be obtained by review of delivery and newborn medical records. Maternal outcomes include date/time of delivery, gestational diabetes, preeclampsia, mode of delivery, indication for operative delivery, and shoulder dystocia. Neonatal, outcomes include hypoglycemia, NICU admission, fracture or nerve injury, fetal anomalies, and breastfeeding upon discharge.

Infant anthopometric measurements. Length will be measured on a hard-surface measuring board; weight, and head circumference measurements will be performed by a trained research team member at approximately 24 hours to 1 week of life. Neonatal skin fold thickness will be measured by a trained research team member at approximately 24 hours to 1 week of life. Length, weight, blood pressure, and skin fold thickness will also be performed by a trained research team member at approximately 48-56 weeks of life. A standardized in-person training will be conducted for the infant skin fold measurements.

Infant Body Composition. (SHARED) Infant body composition will also be measured using air-displacement plethysmography (PEA POD), at parturition and between postpartum weeks 48 and 56.

Breastfeeding will be measured using three questions adapted from the Southampton Women's Survey[14]and CDC Infant Feeding Practices study.[15] The questions assess initiation and duration of breastfeeding, [15] duration of exclusive breastfeeding,[14] and timing of introduction of water and complementary fluids.[14] The questions will be administered between postpartum weeks 48 and 56.

Infant Feeding Styles (SHARED) will be assessed at postpartum weeks 48-56. The 16 item questionnaire, adapated from Thompson et al.[16], assesses Restrictive Feeding Style (3 items - item #: 7, 9, 15), Pressuring/Overfeeding Style (7 items - item #: 1, 2, 4, 6, 10, 11, 14), Responsive Feeding Style (1 item - item #: 5), and Beliefs in the Benefits of Breastfeeding (5 items - item #: 3, 8, 12, 13, 16).

The infant Food Frequency Questionnaire (FFQ) (SHARED) Long-form is a dietary assessment instrument to characterize infants' food intake during the first year of life. The long FFQ was assembled by Life-Moms based on a published shorter version[17], expanding the 19 categories to include 54 food items with a brief description on how these were prepared and/or their source (e.g., raw, canned, etc.). Previous infant studies were used to identify these food items, with particular emphasis to include typical Hispanic foods. It also includes information on supplements use. A food database will be created to calculate nutrient intake for the long FFQ. The long FFQ is self-administered and is a pen-and-paper instrument.

Blood and urine. Maternal blood and urine will be collected at 35-36[6] weeks' gestation and 48-56 weeks postpartum. Samples will be collected in the same manner as the baseline visit.

Achievement of Infant Milestones. Assessing physical activity in infancy is an important objective for lifestyle intervention studies such as LIFE-Moms, as it is possible that infant activity levels track into childhood and impact growth and other health outcomes, and it is unknown if intervening in the mothers during pregnancy impacts infant activity levels. Infants, motor milestones can be quantified easily and may reflect an infant's level of physical activity[18].

In LIFE-Moms, mothers will complete a questionnaire to discern infant motor milestones at the month 12 postpartum visit. These questions are: i) When did your child first roll over without assistance? ii) When did your child first sit up without support? iii) When did your child first crawl on his/her hands and knees? iv) When did your child first walk without assistance?

Child television viewing. Child television viewing will be assessed 12 months postpartum by the following question, which was adapted from another study[19], "Over the past week, how much total time would you say your child spent watching television or videos (including use of Cable, VCR, DVD, computers, mobile phones, and electronic tablets such as iPads)?" Hours of TV viewing will be reported in whole numbers, i.e., 1.0, 1.25, 2.5, etc.

Maternal Body Composition. Maternal body composition will be measured using air-displacement plethysmography, a well-validated technique to measure body composition that utilizes basic gas laws to describe the inverse relation between pressure and volume in two enclosed chambers. The assessment involves the mother sitting quietly in an air displacement plethysmograph (BOD POD), an egg-shaped device, for approximately 10 minutes and forcefully breathing out the air in her lungs 2-3 times, while removing her shirt and wearing shorts and a hair cap (that we will provide). This assessment will be done between postpartum weeks 48 and 56.

Additional study procedures and questionnaires will be site specific.

6.5 Patient Management and Follow-up

Study staff are encouraged to maintain contact with all participants in both groups. The level of contact with the control group depends on the protocol.

6.6 Safety Monitoring / Adverse Event Reporting

The following findings would generate a Core safety alert and specific action:

1. High Score on the Beck Depression Inventory II
   - Actively suicidal
     - BDI-II question 9 value of 2 or 3 requires notification of the Principal Investigator (PI) or a designated clinician. The research staff should wait with the patient until the PI or clinician arrives to handle the case. The PI or designated clinician should assess the patient for risk of imminent harm to self and contact emergency services if needed. If the PI or designated clinician is not immediately available, emergency services should be notified (911).
   - Severe depression
     - BDI-II score of 29-63 requires that research staff notify the PI or designated clinician who should assess the patient for safety and/or possible psychiatric referral. The patient's obstetrician and primary care doctor should also be informed.
   - Moderate depression
     - BDI-II score of 20-28 requires that the participant be informed and that it is recommended she see her physician for further evaluation.
2. Contraindications to aerobic exercise during pregnancy
   - If any of the following are reported, any aerobic exercise portion of the intervention will be stopped. Participants should be instructed that if her doctor limits activity (rest, bedrest, pelvic rest) at any time, she should contact research staff immediately.

- o Incompetent cervix/cerclage
- o Persistent 2nd or 3rd trimester bleeding
- o Placenta previa after 26 weeks' gestation
- o Premature labor that requires hospitalization and treatment with medications to stop contractions
- o Ruptured membranes
- o Preeclampsia / gestational hypertension
- o Severe anemia defined as a hemoglobin < 8
- o IUGR defined as <5th percentile with abnormal Uterine Artery Doppler studies

3. High Blood pressure
   - If measured blood pressure is ≥ 160/110, study staff will call the participant's provider immediately and have a plan in place before the patient is allowed to leave.
   - If measured blood pressure is ≥ 140/90, study staff will notify participant's provider
4. Weight loss from enrollment weight during pregnancy
   - Alert values will be weight loss of 4% or greater of enrollment weight for women who were overweight at enrollment and loss of 6% or greater of enrollment weight for women who were obese at enrollment.
   - For the first alert, the research staff will notify the patient, discuss her energy balance activities, provide weight management counseling, and modify the intervention if appropriate. Participants with a first alert for weight loss will return for a study visit weight within 2 weeks.
   - If the weight loss is the same or greater upon reassessment, the patient's physician should be contacted and the intervention may need to be modified (i.e. decrease exercise, increase caloric intake). If the weight upon reassessment is no longer at alert values, the participant will continue current study activity.
5. Incidental findings
   - When incidental findings on imaging studies or out of range values on lab tests are obtained by study personnel, the participant will be notified and a copy of the report sent to her physician. For lab tests, this pertains only to those tests for which results are obtained in real-time.

Sites may choose to have additional safety alerts as defined within their protocol.

6.6.1 Definitions of AE/SAE

An **adverse event** is any untoward medical occurrence associated with participation in the study.

- Musculoskeletal injury that requires medical attention
- Fetal growth restriction (<5th percentile with abnormal umbilical artery Dopplers)
- Unanticipated untoward medical events that are not plausibly pregnancy related and may be study related

A **serious adverse event** is defined as an untoward medical occurrence, whether associated with study participation or not, that results in one of the following:

- Death
    - Maternal death
    - Fetal or infant death, including miscarriage, therapeutic abortion because of increasing signs of maternal or fetal compromise, and stillbirth.
- Life-threatening event
    - Life threatening events in the mother, fetus, neonate or infant are defined as those that in the view of the research staff and PI put the individual patient at imminent substantial risk of dying, or if continued participation in the study might have resulted in death.
- Hospitalization (initial or prolonged)
    - Maternal hospitalization alone is not sufficient to qualify as a serious adverse event. Hospitalization for the following would not be considered a serious adverse event: delivery, preterm premature rupture of membranes (pPROM), pylonephritis, bedrest, contractions, and preterm labor.
    - Infant or child re-hospitalization after neonatal discharge may be reported as a serious adverse event.
    - Any medical or surgical procedure performed (e.g., surgery, transfusion) itself is not the adverse event; instead, the condition that leads to the procedure is the adverse event.
    - Emergency room visits that do not result in admission to the hospital should be evaluated for one of the other serious outcomes (e.g., life-threatening; required intervention to prevent permanent impairment or damage; other serious medically important event). Emergency room visits for the following would not be considered a serious adverse event: pylonephritis, contractions, and preterm labor.
- Disability or permanent damage
    - If the adverse event resulted in a substantial disruption of the mother or infant's ability to conduct normal life functions, i.e., the adverse event resulted in a significant, persistent or permanent change, impairment, damage or disruption in the patient's body function/structure, physical activities and/or quality of life. The recommendation for bedrest does not constitute an SAE.
- Congenital anomaly /birth defect
- Intervention to prevent permanent impairment or damage

Other serious important medical events also qualify. When an event does not fit the other outcomes, but may jeopardize the patient and may require medical or surgical intervention or treatment to prevent one of the other outcomes, this should also be reported as an SAE.

6.6.2 Rules for Intervention Termination / Modification

Each protocol describes situations in which the intervention may be modified or terminated (i.e. if diagnosed with Gestational Diabetes or activity restriction).

6.7 Study Outcome Measures and Ascertainment

6.7.1 Primary Outcome

The primary outcome is defined as gestational weight gain above the 2009 goals established by the IOM. For the purpose of this primary outcome, gestational weight gain is defined as the difference between the maternal weight measured at the 36 week visit and the baseline weight.

6.7.2 Secondary Outcomes

Secondary analyses will compare the effect of the intervention compared to control condition on the following secondary outcomes in both the mother and off-spring. As relevant, measures will be evaluated serially throughout the intervention and follow-up periods. Sub-group analyses (including race/ethnicity, age, parity, baseline weight, and gestational weight gain) will be performed on all maternal and offspring secondary outcomes. Outcomes (other than gestational weight gain) will also be assessed according to those who met vs. exceeded IOM-recommended gestational weight gain guidelines. Unless otherwise specified, all maternal repeated measures analyses will include baseline, 24-26 week, 36 week and 1-year post-partum data elements.

6.7.3 Maternal Secondary Outcomes

1. Gestational weight gain per week
2. Occurrence of GDM defined by the 75g OGTT, OGTT glucose measurements
3. Second trimester weight gain defined as baseline to 24-26 week measured weight.
4. Weight gain starting from self-reported pre-pregnancy weight
5. Metabolic profiles (glucose, insulin, lipids, and hsCRP) and blood pressure
6. Physical activity assessment and sedentary time
7. Gestational hypertension, preeclampsia
8. Induction of labor
9. Mode of delivery
10. Preterm delivery (spontaneous versus indicated)
11. Shoulder dystocia
12. Post partum weight retention
13. 12 months post-partum pre-diabetes
14. 12 months post-partum diabetes

6.7.4 Fetal and Neonatal Secondary Outcomes

1. Gestational age at delivery
2. Birth weight,
3. Large for gestational age (Alexander)
4. Small for gestational age
5. Macrosomia (≥4000 g)
6. Birth length
7. Head circumference
8. Birth trauma
9. Neonatal intensive care unit admission
10. Respiratory morbidity

6.7.5 Infant Secondary Outcomes

1. Anthropometrics including length, weight, head circumference, skinfolds
2. Blood pressure at 12 months

7 Statistical Considerations

7.1 Analysis Plan

7.1.1 Methodology

- Baseline weight: measured weight at randomization minus an adjustment for women randomized at 14 or 15 weeks (-1 lb for women at 14 weeks, - 2 lbs for women at 15 weeks). No adjustment will be made for women randomized between 9 and 13 weeks.
- Baseline BMI will be based on baseline weight and measured height
- Ending weight: study measured weight at 36 weeks. If the patient delivers before the 36 week measure is done, the last recorded clinic measured weight will be used.
- Gestational weight gain will be defined as (ending weight – adjusted starting weight)/ time elapsed in days/7 i.e. weight gain per week.
- Weight gain per week will be compared with IOM guidelines weight gain per week in the second/third trimester for overweight and obese defined by baseline BMI defined above
- If a patient has a first trimester miscarriage, the outcome will be set to 'not excessive weight gain'
- All patients will be included in the primary analysis unless they are lost to follow-up
- Lost to follow-up is defined as no weight measurement (either in clinic or by study measurement) beyond the randomization weight, unless the patient had a first trimester (<14 weeks 0 days) miscarriage

7.1.2 Handling Missing or Incomplete Data

The first strategy will be to prevent the occurrence of missing outcome data. Missing pregnancy outcome data (including maternal weight gain) as well as missing neonatal outcomes derived from the medical charts are expected to be very low. Women who plan to deliver at a hospital outside of the study center will be excluded before randomization. All women will be required to sign a medical records release for themselves and their newborn prior to randomization in case delivery does occur elsewhere. Multiple methods for contacting women will be sought at the initial interviews, including contact information for friends and relatives. Loss to follow up and missing and incomplete data will be monitored closely by the Design, Data Quality and Data Analysis Committee of the Consortium as well as the DSMB, to solve potential issues of missing data before there is a substantial impact on the results.

For data collected as responses to questionnaires, all forms will be reviewed for completeness before the participant leaves the study visit. Data entry will be accomplished quickly so that missing values may still be retrieved. For weight gain and related outcome variables in particular, if the study measured weight from the scheduled third trimester study visit at 35-36 weeks is not available, the closest maternity care provider visit weight (preferably on or before 36 weeks) will be used.

Under intention-to-treat principles, all participants will be included in the analysis. Missing data related to the primary outcome will be evaluated to assess whether the missing mechanism may be ignorable or non-ignorable[20-22]. If the missing data mechanism is judged to be ignorable, where appropriate, analyses involving mixed models may be used such that all existing values are analyzed, and no observations are deleted due to missing values. Alternatively multiple imputation may be carried out to create several complete data sets. For each complete data set, overall tests of interest for the outcome will be conducted and the results of each combined to create a single test result. For completeness, a pattern-missing analysis should be conducted to investigate non-ignorable missingness. If the missing data mechanism is likely to be non-ignorable, multiple imputations can be conducted using a version of the approximate Bayesian bootstrap based on distance-based selection criteria[23]. Sensitivity analyses under various assumptions regarding the missing data will be conducted to confirm the robustness of the results.

8 Data Collection

8.1 Data Collection Forms

Data will be collected on standardized forms on which nearly all responses have been pre-coded. Each form is briefly described below:

- LM01 Randomization Form is completed for all randomized patients and records project gestational age, other eligibility criteria, and a randomization code.
- LM02 Baseline Form is completed for all randomized patients. This form includes detailed demographic and social data, medical and obstetrical history, and current pregnancy complications.
- LM02A Previous Pregnancy Outcome Form
- LM03A Study Visit Form is completed between 24,0 weeks and 27,6 weeks gestation.
- LM03B Study Visit Form is completed between 35,0 weeks and 36,6 weeks gestation.
- LM04 Labor and Delivery Summary Form documents basic delivery information such as date/time of delivery, mode of delivery, type of labor, and presence of shoulder dystocia.
- LM05 Neonatal Baseline Form records birth weight, length, head circumference, skin fold measurements, and neonatal morbidity.
- LM06 Patient Status Form documents loss to follow up/ withdrawal status/reason, and last date of contact for lost to follow-up patients.
- LM07 Adverse Event Form records adverse events.
- LM08 Follow-up Visit Form for the 12 month follow-up visit.

- Participant Questionnaires:
    - BDI-II The commercially purchased BDI-II form will be performed at baseline as part of the Core exclusion criteria and at other timepoints specific to the site's protocol.
    - Sleep Questionnaire will be used to access sleep schedule, quantity, quality, habits, disorders, and sleepiness.

8.2 Data Entry and Management System

8.2.1 Quality Control

Data checks are produced weekly by the RCU. Data checks include dictionary edits for missing or "out of range" values, consistency checks which examine the relationship between two or more questions on the same form, and audit checks which compare multiple questions across different forms.

All of these data checks are examined by the research assistant at the RCU to see whether a valid "override" comment (and/or nurse's initials) that may explain the data has been keyed. Data checks with a valid comment or verification are suppressed and never sent back to the centers. Data checks for which no response or correction has been received by the end of the month are re-sent at the beginning of the next month. Data checks are available for access on MIDAS on Friday mornings, with some exceptions around the holidays.

Weekly, bi-weekly, and/or monthly checks of data, outside the normal data check routines, are conducted for some studies. These might, for example, include checks for eligibility and timeliness of data form entry.

Data quality reports are generated for semi-annual Steering Committee meetings and are based on the following measurements:

- Number of data checks per 1000 fields keyed
- Number of outstanding data checks
- % Overdue and late forms
- Depending on specific study parameters, issues related to protocol “violations” and protocol “deviations” are also included in data quality reports.

8.2.2 Confidentiality and Data Security

Site research staff will transcribe data obtained from the subject's medical record, or by interview or from observation of study procedures on to case report forms. These forms will include the unique identifier described above, so that the case report forms from different subjects are correctly assigned. These completed case report forms, where applicable, for a given subject will be kept at the site, along with portions of the medical chart as needed (for example a copy of an ultrasound report needed to establish gestational age) in a patient research folder in the locked filing cabinet. The research staff at the clinical center will enter the data from the case report forms or upload files via secure FTP into the web data management system which is housed by The GWU Biostatistics Center. The data are identified only by the unique identifier. No identifiers, such as name, address, social security number, or physician’s name are entered on the web data entry system. SSL (Secure Socket Layer) security and encryption algorithms are used when transmitting or receiving data to and from the clinical centers. Data from the web data management system are uploaded into SAS datasets for data analysis on to the mainframe servers. The servers are all inside a secure walled data room which is protected by a combination lock entry system and surveillance cameras. Access to the room is restricted to authorized systems staff. Access to the data at GWU Biostatistcs Center is secured by user accounts and passwords specific to each employee and will be restricted only to those staff working on this particular trial. Wireless access to the network that houses the research data is not allowed. The network is protected from outside networks and the Internet by way of an active firewall and various filtering rules.

Copies of portions of medical charts may be required for verification of Core/Shared data. These are carefully de-identified by the clinical center before mailing by blacking out any identifiers such as name or hospital number, and photocopying the chart for mailing. All sites are trained in de-identification procedures before the start of the study.

Individual study participant data may be presented in reports prepared for the site investigators, for the purpose of data quality monitoring but never in publications. At the conclusion of the trial (no later than two years after the final visit/data collection) a dataset will be prepared for the sites and for public access as part of the NIH requirement for data sharing. For this dataset the identifiers will be removed and the order of the patients will be scrambled. Variables with low frequencies for some values that could potentially identify a participant will be recoded into categories.

8.2.3 Sharing of Data for Common Measures

All core data will be entered directly into a MySQL database maintained by the RCU either through direct data entry using the RCU's web-based data management system (MIDAS) or by file upload via secure FTP. The RCU is responsible for quality control of the database and will generate data queries both at the point of entry and in batch, as well as additional checks for data consistency within or across forms.

At the completion of the project, all core data will be transferred by the RCU to the NIDDK Repository. In doing so measures will be taken to protect patient confidentiality, including replacing the patient ID, scrambling records out of order, removing dates and replacing them with days elapsed since randomization, and recoding variables with low frequencies into categories, or combining categorical variables.

8.2.4 Transfer of Center-Specific Data to NIDDK Repository

At the completion of the trial, center-specific data that are not entered into the RCU system will also be transferred to the NIDDK repository. Similar methods will be used to protect patient confidentiality.

8.3 Performance Monitoring

The RCU will present regular reports to the LIFE-Moms Steering Committee, Safety subcommittee, and the Data and Safety Monitoring Board. These include:

- Monthly Adverse Event Reports – Adverse Event reports will be tabulated and sent to the Safety Committee for review.
- Semi-annual Steering Committee Reports – reports detailing recruitment, baseline patient characteristics, data quality, incidence of missing data and adherence to study protocol by sites, are provided twice per year to all members of the Steering Committee. These reports will be run in conjunction with the Data and Safety Monitoring Board reports.
- Data and Safety Monitoring Board Reports – for every meeting/call of the DSMB, a report, for all seven protocols, is prepared on safety issues, recruitment, retention, protocol adherence and data quality and timeliness. Core measures and data may be compared across the seven sites.

9 Study Timetable

9.1 Training and Certification

The following happened during Year 1 of the Consortium:

- November 2011 – Initial welcome webinar
- January 2012 – First Steering Committee
- February / March 2012 – Subcommittees determined/proposed Core measures
- March 2012 – Second Steering Committee to decide on Core measures
- January / May 2012 Protocols completed and reviewed by NIH/RCU
- June 2012 – First DSMB meeting
- August-October 2012 – Central training for maternal circumferences, maternal skin folds, infant skin folds, & BDI-II administration.

9.2 Recruitment and Data Collection Period

Recruitment is planned to commence in September 2012. The recruitment table allows for approximately 7 months until delivery (12 weeks – 40 weeks) and 12 months until the follow-up visit (48-56 weeks postpartum). The table lists cumulative numbers of participants per quarter as described in each of the site's protocols. Completed refers to competing the 12 month postpartum visit.

	Year 2 - Sept12-Aug13				Year 3 - Sept13-Aug14				Year 4 - Sept14-Aug15				Year 5 - Sept15-Aug16			
	Q1	Q2	Q3	Q4	Q1	Q2	Q3	Q4	Q1	Q2	Q3	Q4	Q1	Q2	Q3	Q4
CP/B - Recruited	39	78	117	156	195	234	273	312	350							
CP/B - Completed							39	78	117	156	195	234	273	312	350	
St. Lukes - Recruited	25	49	75	103	129	157	183	210								
St. Lukes - Completed							25	49	75	103	129	157	183	210		
UPR - Recruited		57	114	171	228	285	342	400								
UPR - Completed							57	114	171	228	285	342	400			
NW - Recruited	43	86	129	172	215	258	300									
NW - Completed							43	86	129	172	215	258	300			
WU - Recruited	33	63	93	123	153	183	213	243								
WU - Completed							33	63	93	123	153	183	213	243		
PBRC - Recruited	36	72	108	144	180	216	252	288	306							
PBRC - Completed							36	72	108	144	180	216	252	288	306	
PIMC - Recruited	15	33	53	74	95	116	137	158	179	200						
PIMC - Completed							15	33	53	74	95	116	137	158	179	200

Note: Q1 denotes September 1-November 30, Q2 notes December 1-February 28, Q3 notes March 1-May 31, and Q4 denotes June 1-August 31

All participants will be recruited by the end of Q3, Year 4. The last visit for the last participant is anticipated to occur in Q4 of year 5.

9.3 Final Analysis

After a two-month period for completion of data entry for the trial and close-out, the data set will be locked and available for analysis. Approximately six months will be required to complete the final report to the Steering Committee and to submit the study's primary report for publication.

Appendix A. Intervention by Component

Center	Dietary Intervention	Physical Activity Intervention	Contact Interval	Other
**Cal Poly/ Rhode Island (1)**	Dietary prescription<br>• Weight gain goals based on IOM guidelines<br>• Specific calorie (25 kcal/kg) and fat goals (<30%)<br>• Weekly behavioral goals (challenge cards) via mail<br><br>Partial meal replacement (PMR) program<br>• PMR 2 meals per day<br>• Participants should consume 1 meal of regular foods and 2-4 healthy snacks each day<br><br>Behavioral (e.g., self monitoring, stimulus control), and home environment goals	Participants will be encouraged to aim for a goal of 30 minutes of activity (e.g. walking, swimming, light aerobic dancing) on most days of the week.<br><br>Weekly educational tips via mail<br><br>Behavioral and home environment goals<br><br>Participants will be provided with a pedometer and advised to gradually increase the number of steps walked each day.<br>• Each week the goal will be to achieve 500 steps more per day (1/2 mile) until reaching an ultimate goal of 10,000 steps/day.	• 1 -15 minutes, face-to-face counseling session for control patients.<br>• 2 -30 minute, face-to-face counseling sessions at the onset of treatment (2 weeks apart)<br>• Face-to-face counseling sessions 2/month until 20 wks gestation; then 1/month until delivery (change back to 2/month for women exceeding or underweight goals)<br>• Mailed weekly educational/ behavior change materials<br>• Mailed quarterly study newsletters (all participants)<br>• Face-to-face counseling sessions at 6 and 12 months postpartum<br>• Assessments at study entry (10-16 weeks), 30 weeks, delivery, 6, and 12 months postpartum<br>• Anthropometrics at delivery and 2 months postpartum	Postpartum diet and education<br>• PRM 1 meal per day<br>• Guidelines for maintaining a healthy home food/exercise
**St. Luke's/Roosevelt Hospital (2)**	Individual flexible counseling on behavior and nutrition<br>• Caloric/nutritional intake based on pre-pregnacy BMI, mypyramid.gov, and ADA meal plans<br>• Physical activity (PA) guidelines based on ACOG recommendations<br>• PA and caloric guidelines	Intervention participants receive individual flexible counseling on exercise<br>• Increase caloric expenditure by at least 700 kcal/week through moderate exercise (walking, swimming, etc.)<br><br>Control participants will be given ACOG recommendations at their 20	• 1 -20 minute individual followed by group meetings every 8 weeks, for control participants<br>• 1 -1 hour introductory individual session for intervention participants followed by individual counseling sessions weekly<br>▪ Pre-natal groups as needed<br>• Email/telephone counseling 2 additional times per week	Intervention postpartum diet and exercise groups will be offered as needed<br>• 3 different group class times will be offered for each topic<br>• Increase caloric expenditure by at

Center	Dietary Intervention	Physical Activity Intervention	Contact Interval	Other
**St. Luke's/Roosevelt Hospital (2) continued**	adjusted throughout the pregnancy<br><br>Food records are used to promote awareness of diet and activity patterns<br><br>Weight charts are used to show recommended weight gain and track the patient's weight	minute counseling session and counseled to increase physical activity to at least 30 minutes per day, five days per week	• A password protected internet message board for participants to question counselors or other participants	least 700 kcal/week through moderate exercise (walking, swimming, etc.)
**University of Puerto Rico (3)**	• Nutritional intervention based on total calorie intake and diet quality including recommending appropriate supplements<br>• Provide healthier food items including:<br>▪ brown rice (enough for 2 servings per day)<br>▪ case of bottled water<br>▪ bottle of vegetable oil<br>▪ bottle of vegetable spread low in saturated fat<br>**Infants**<br>Parents of intervention participants receive nutrition education (encouraging breastfeeding, preventing overfeeding and promoting healthy food choices and feeding styles, recommending supplements.	Increase non-exercise activity thermogenesis (NEAT)<br>• Daily self-monitoring of daily step counts using a pedometer<br>• Reducing automation (stairs versus escalators)<br>• Interrupting periods of sitting time with physical activity such as standing and walking<br><br>**Infants**<br>Education component to the mother about how she can physically activate her baby by age and ability appropriate activities (i.e. hand clapping, assisted standing, back turns and rolling, toe touching, reaching, and active play).	• Face-to-face intervention visit focusing on caloric plan and individualized physical activity prescription<br><br>• 5-1/2 day, group Pregnancy empowerment workshops<br>▪ Groups of 8-12 women, with similar due dates, take the courses together<br>▪ Including a cooking demonstration and group activities<br><br>• Centering Pregnancy Group Prenatal visits<br>• Groups are every 4 weeks for 4 sessions, then every 2 weeks for 6 sessions with reinforcement of diet and physical activity recommendations<br><br>• Monthly follow-up phone calls (prenatally through the first year postpartum)	Empowerment intervention, Centering Pregnancy group prenatal care

Center	Dietary Intervention	Physical Activity Intervention	Contact Interval	Other
**University of Puerto Rico (3) continued**			**Infants**<br>• Face-to-face visit at 4 – 6 weeks and 16 – 24 weeks with mothers and infants<br>• Monthly follow-up phone calls with mothers	
**Northwestern (4)**	**Control participants**:<br>receive educational advice to follow the USDA MyPlate.gov and US Dietary guidelines<br><br>**Intervention participants:**<br>receive:<br>• Calorie-controlled, individually tailored DASH diet to optimize the nutrition quality of the maternal diet throughout the study and breast milk postnatally.<br>• Emphasis on a nutrient-rich, calorie-controlled diet to minimize weight gain prenatally and maximize weight loss diet postpartum.<br>• CalorieKing diet software for self-monitoring to ensure daily calorie/nutrient goals are met.<br>• Breastfeeding will be strongly encouraged early in the prenatal period and appropriate support provided to maximize duration of BF postpartum.	**Control participants**:<br>will not be provided any recommendations for physical activity.<br><br>**Intervention participants:**<br>receive the ACOG recommendations<br>• $\geq$30 minutes of moderate-intensity activity 5-7 times per week.<br>• Pedometers and exercise logs will be provided to help participants meet their exercise goals.<br><br>Postpartum: new exercise goals will be individualized as appropriate	**Control participants:**<br>Quarterly face-to-face groups (2 prenatal; 4 post-partum) covering infant CPR, care for newborns and transitions to motherhood<br><br>**Intervention participants:**<br>• Emailed weekly newsletter: general well care (both groups)<br>• Intervention:<br>1. 7 face-to-face group sessions (6 prenatal; 1 postpartum) for intervention participants<br>2. 5 face-to-face/phone individual sessions with RD (3 prenatal; 2 postpartum)<br>3. Telephone counseling (behavior coach)<br>▪ Weekly (first 6 weeks)<br>▪ Monthly (4 months prenatal through - 11 months postpartum)<br>4. Weekly emails/texts regarding Calorie King self-monitoring of diet/ weight/ physical activity (24 months – intervention group)	

Center	Dietary Intervention	Physical Activity Intervention	Contact Interval	Other
**Northwestern (4) continued**	• Parenting skill training postpartum to promote healthy infant feeding and activity behaviors.		5. Educational modules (PDFs) emailed<br>▪ 5x prenatally; 8 x post-partum for intervention participants	
**Washington University (5)**	Intervention participants will receive standard prenatal care, the Parents as Teachers (PAT) program, and a diet and exercise intervention program integrated within a home visiting curriculum delivered during pregnancy through 18 months post-partum.<br><br>Content and strategies are organized around social cognitive behavior change theory and will target: a) goals and strategies for achieving appropriate GWG in pregnancy, b) substituting water or low-fat milk for high-sugar beverages, c) substituting healthy foods for unhealthy foods, d) portion control, e) strategies for increasing access and availability of nutritious foods in the home, f) regular eating patterns (3 meals and 1-2 snacks per day), g) dealing with food cravings, and h) breastfeeding barriers and strategies.	Changes in physical activity patterns will be designed to increase daily energy expenditure through walking with an ultimate goal of progressing to a total of 150 minutes (2.5 hours) of moderate-intensity exercise (e.g. brisk walking) and/or lifestyle activity per week.<br><br>Physical activity goals may be modified as needed for participants with higher and lower exercise tolerance.<br><br>Intervention participants will receive a "Smart" Pedometer, which allows them to upload their steps to a website using a username and password.	• PAT program<br>▪ 10 weekly/biweekly prenatal home visits<br>▪ 18 monthly postpartum home visits<br>• Face-to-face check-in visit at the hospital for intervention participants<br>• Website access for intervention participants<br>▪ Log steps<br>▪ Set goals<br>▪ Track progress towards goals<br>▪ Create graphs/charts<br>▪ Log other activities<br>▪ Log weight	Postpartum diet and education<br>• Reinforce lessons learned in the prenatal phase to reduce weight retention and the goal of returning to pre-pregnancy weight<br>• Positive child-feeding behaviors<br>• Interactive parent-child activities

Center	Dietary Intervention	Physical Activity Intervention	Contact Interval	Other
**Pennington (6)**	Dietary intake recommendations are established in accordance with the 2010 USDA Dietary Guidelines for Americans. The energy intake target is designed to keep the participant within the IOM GWG guidelines.<br><br>18 lessons and topic areas given in either a clinic setting or by phone	Exercise recommendations include 150 minutes/week of moderate intensity activity.<br><br>3-4,000 steps per day above baseline<br><br>18 lessons and topic areas given in either a clinic setting or by phone	SmartMoms-Clinic group participants will attend:<br>First 12 weeks (2nd tri)<br>• 3 group sessions / month<br>• 1 individual session /mth<br>Week 25 – Birth<br>• 1 group session / month<br>• 1 individual session / mth<br><br>SmartMoms-Phone group participants will receive:<br>• 2 individual sessions (enrollment and last session)<br>• All other sessions will be delivered via an interactive Smartphone app. Discussion with counselor as needed/ directed by toolbox<br>• Body Trace scale to transmit their body weight to the counselor daily<br>• Stepcounter to transmit stepcount daily to the counselor<br>• Body wt. and step data will be used to guide treatment recommendations	

<table>
<tr><th>Center</th><th>Dietary Intervention</th><th>Physical Activity Intervention</th><th>Contact Interval</th><th>Other</th></tr>
<tr><td>NIDDK- PIMC (7)</td><td>Individualized weight gain goals based on IOM recommendations modified to match the lower overall pregnancy weight gain goals graphed for weight tracking.

Caloric recommendations based on 70% DRI. Fat intake <30% total fat calories per day.
• Individualized meal plan (4-6 portion controlled meals/snacks)
• Instructions for measuring, weighing, and self-monitoring food intake
• Feedback
• Self-weighing

Problem solving</td><td>Daily physical activity such as walking, reducing use of automation (for example taking stairs instead of elevators)

Reducing time spent in sedentary behaviors such as sitting or lying

Increase non-exercise activity thermogenensis
• Daily self-monitoring logs

Participants who do not meet weight gain guidelines or who are unable to achieve dietary or physical activity goals may receive the following:
• Once or twice weekly supervised exercise training at fitness centers or parks
• Unsupervised exercise options</td><td>Intervention:
• 4 initial individual sessions
• Weekly group sessions
• 1 monthly midwife centering Participants may bring a support person.

Standard of care:
• 3 sessions over pregnancy in group or individually

Postpartum both groups:
• 3 groups or one on ones; infant feeding practice, parenting, and ways to promote family physical activity</td><td></td></tr>
</table>

References

1. Fairburn CG, Berglin SJ. Assessment of eating disorders: Interview or self-report questionnaire? Int J Eat Disord 1994;6:1-8.

2. Beck, A.T., R.A. Steer, and G.K. Brown, BDI-II Manual. 1996, San Antonio, TX: Harcourt Brace & Company.

3. Lacasse A, Rey E, Ferreira E, Morin C, Bérard A. Validity of a modified Pregnancy-Unique Quantification of Emesis and Nausea (PUQE)scoring index to assess severity of nausea and vomiting of pregnancy. Am J Obstet Gynecol 2008;198:71.e1-71.e7.

4. Wing RR, Tate DF, Gorin AA, et al. STOP regain: are there negative effects of daily weighing? *J Consult Clin Psychol.* 2007;75(4):652-656.

5. Phelan S, Liu T, Gorin A, et al. What distinguishes weight-loss maintainers from the treatment-seeking obese? Analysis of environmental, behavioral, and psychosocial variables in diverse populations. *Ann Behav Med.* Oct 2009;38(2):94-104.

6. Ware JE, Jr., Kosinski M, Keller SD. A 12 Item Short Form Health Survey: Construction of scales and preliminary tests of reliability and validity. *Med Care* 1996; 34:220-233.

7. Brown, K. W., Ryan, RM. The benefits of being present: Mindfulness and its role in psychological well-being. J Pers Soc Psychol. 2003; 84:822-848

8. Kabat-Zinn, J. Massion, A, Kristellar, J, Peterson, L G, Fletcher, K Pbert, L, Lendrking, W. Santorelli, S. Effectivenss of a meditation based stress reduction program. Am J Psychiatry. 1992; 149:936-943.

9. Kabat-Zinn, J. Lipworth, L, Burney, R. The clinical use of mindfulness meditation for the self-regulation of chronic pain. J Behav Med. 1985; 8:163-190.

10. Melbourne Academic Mindfulness Interest Group. Mindfulness-based psychotherapies. A review of conceptual foundations, empirical evidence and practical considerations. Aust NZ J Psychiatry. 2006; 40:285-294.

11. Framson, C, Kristal, A, Schenk, J, Littman A, Zeladt, S and Bentez, D. Development and validation of the Mindful Eating Questionnaire. 2009; Journal of the American Dietetic Association; 109:1439-1444.

12. Sallis JF, Kerr J, Carlson JA, et al. Evaluating a brief self-report measure of neighborhood environments for physical activity research and surveillance: Physical Activity Neighborhood Environment Scale (PANES). Journal of physical activity & health 2010;7:533-40.

13. Automated Self-Administered 24-Hour Dietary Recall (ASA24), Version 1 (2011). Bethesda, MD; National Cancer Institute.

14. Marriott LD, Inskip HM, Borland SE, et al. What do babies eat? Evaluation of a food frequency questionnaire to assess the diets of infants aged 12 months. *Pub Health Nutr.* 2008:1-6.

15. Fein SB, Labiner-Wolfe J, Shealy KR, et al. Infant Feeding Practices Study II: study methods. *Pediatrics.* Oct 2008;122 Suppl 2:S28-35.

16. Thompson AL, Mendez MA, Borja JB, Adair LS, Zimmer CR, Bentley ME. Development and validation of the Infant Feeding Style Questionnaire. Appetite 2009;53:210-21.

17. Grummer-Strawn, Laurence M., Kelley S. Scanlon, Sara B. Fein. 2008. Infant Feeding and Feeding Transitions during the First Year of Life. *Pediatrics*: 122(suppl 2): S36-S42.

18. Benjamin Neelon SE, Oken E, Taveras EM, Rifas-Shiman SL, Gillman MW. Age of Achievement of Gross Motor Milestones in Infancy and Adiposity at Age 3 Years. Matern Child Health J. 2011 Jun 4.

19. Burdette HL, Whitaker RC. A national study of neighborhood safety, outdoor play, television viewing, and obesity in preschool children. *Pediatrics.* Sep 2005;116(3):657-662.

20. Rubin DB. Multiple . Imputation for Nonresponse in Surveys. John Wiley and Sons, Inc, New York, NY, 1987.

21. Little RJA, Rubin DB. Statistical Analysis of Missing Data (second edition). John Wiley and Sons, Inc, New York, NY, 2002.

22. Schafer, JL. Analysis of Incomplete Multivariate Data. Chapman and Hall/CRC, New York, NY, 2000.

23. Siddique J, Belin TR. Using an approximate Bayesian bootstrap to multiply impute nonignorable missing data. Comput Stat Data Anal 2008; 53: 405-415

Metabolic Health During Pregnancy and a Possible Explanation for Hetrogenous Outcomes To Prenatal Interventions: A Post Hoc Analysis of the LIFE-Moms Randomized Clinical Trial

Writing Group Leads: Emily Flanagan, Leanne Redman
Statistician: Kimberly Drews
Other Writing Group Members: Todd Cade, Paul Franks, Dympna, Gallagher, Suzanne Phelan, and Linda Van Horn

Statistical Analysis Plan

1 Study Overview

The LIFE-Moms consortium was brought together to investigate the impact of lifestyle interventions on controlling gestational weight gain (GWG) in women who were overweight or obese at conception. It was designed as an individual patient meta-analysis using data from seven independent trials all with the common GWG purpose. The main outcome and pre-specified secondary outcomes were identified by the consortium, while additional secondary investigations were proposed by individual investigators within the consortium to investigate other aspects of the data. This statistical analysis plan pertains to one of the investigator-initiated analyses.

The underlying premise to this investigation is that not all obesity is equal. The idea that a certain phenotype of obesity that is 'metabolically' healthy is now widely studied and recognized. The prevalence of 'metabolically healthy' obesity in women entering pregnancy has not been studies nor has the pattern of gestational weight gain, effect on maternal and infant outcomes, or the impact of lifestyle interventions. Although standard criteria to define metabolically healthy obesity does not yet exist, the promise of personalized medicine for targeted therapies (such as lifestyle intervention) underscores the need to consider other metabolic and anthropometric parameters in addition to BMI to define obesity.

In this study, we aim to identify metabolically healthy and metabolically unhealthy obese pregnant women in LIFE-Moms. First in a cross-sectional analysis we will compare the changes in gestational weight gain (total, g/week) and adherence to IOM guidelines (%) in these two groups and the incidence of maternal (GDM, hypertension etc) and infant outcomes (LGA, SGA, macrosomia). Second, we will test if a lifestyle intervention that reduced gestational weight gain overall, is more or less effective in metabolically unhealthy obese pregnant women with regard to adherence to IOM guidelines and maternal and infant outcomes.

2 Study Population

All women who were members of the LIFE-Moms cohort who were obese (BMI ≥ 30 kg/m^2) were eligible for inclusion in this analysis. Those included in the main analysis were either those who were metabolically healthy or metabolically unhealthy (defined below). All infants of the mothers for whom we had data were included in the analysis of neonatal outcomes.

3 Outcomes, Exposures, and Additional Variables of Interest

3.1 Outcomes

The analysis focuses on pregnancy, delivery, and neonatal outcomes in the eligible subcohort from LIFE-Moms. The outcomes are divided into two categories: previously defined by the main study and newly defined for this manuscript.

3.1.1 Previously Defined Outcomes

Many of the outcomes examined in this project were defined at the study level and were not redefined for this analysis. The definitions of these outcomes can be found in the overarching study statistical analysis plan.

These outcomes include: all gestational weight gain variables, gestational diabetes, gestational hypertension, preeclampsia, cesarean delivery, preterm delivery, birth weight, and sum of skinfolds.

3.1.2 Newly Defined Outcomes

An additional estimate of neonatal body composition was calculated for this analysis. The calculation of neonatal fat mass utilizes the Catalano et al. equation.[1] Any infant born pre-term will not be included in this calculation as the results have not been validated in these neonates.

3.2 Exposure Variable

Each participant will have a measure of metabolic health calculated using the National Cholesterol Education Program Adult Treatment Panel III guidelines for metabolic risk criteria.[2] Since waist circumference was not measured in all of the LIFE-Moms cohort, this criterion will not be considered in any of the participants. The remaining criteria considered will be systolic blood pressure > 130 mm Hg or diastolic blood pressure > 85 mm Hg; HDL cholesterol < 50 mg/dL; LDL cholesterol ≥ 100 mg/dL; triglycerides ≥ 150 mg/dL; glucose ≥ 100 mg/dL. If a participant meets none of the criteria at baseline they are classified as metabolically healthy obese (MHO) whereas if they meet two or more of the criteria they are classified as metabolically unhealthy obese (MUO). Any participant meeting only one of the criterion has intermediate status and will therefore be excluded from the analysis.

3.3 Baseline Characteristics

All baseline characteristics will be reported as defined in the main study statistical analysis plan.

4 Statistical Analysis Plan

4.1 Demographic and Clinical Characteristics ("Table 1")

Raw means and standard deviations will be presented for continuous variables and percentages will be presented for categorical variables. All results will be presented overall and by metabolic status classification (MHO vs. MUO). Tests for difference in baseline characteristics between treatment groups will be conducted using generalized linear mixed models (GLMM) with a random effect for protocol, given the consortium model of the data. This will allow for covariance structures to be developed within each protocol and between the protocols. The exception to this will be for testing race-ethnicity differences as the inclusion criteria for some of the protocols result in a lack of variability to allow for testing. Metabolic variables will be adjusted for enrollment BMI, race/ethnicity, and maternal age at enrollment.

4.2 Gestational Weight Gain Outcomes

4.2.1 GWG by Metabolic Health Status

Overall gestational weight gain (GWG), GWG for the 2nd and 3rd trimester, as well as classification of whether there was excess GWG for each will be analyzed using GLMM with a random effect for protocol. These analyses will be done using an unadjusted analysis, which only includes treatment assignment as a covariate, as well as an adjusted model which also includes maternal age at enrollment, race/ethnicity, and enrollment BMI as covariates. Summary statistics for all groups will be calculated as either means ± SD or percentages.

4.2.2 GWG by Metabolic Health Status and Randomization Assignment

An interaction term will be added into the model to assess the interrelationship between health status and assignment to lifestyle intervention versus standard of care on GWG outcomes. The models examined will be the same otherwise. Summary statistics for all groups will be calculated as either means ± SD or percentages.

4.3 Pregnancy Outcomes

4.3.1 Pregnancy Outcomes by Metabolic Health Status

The relationship between incidence of gestational diabetes (GDM), gestational hypertension, preeclampsia, cesarean delivery, and preterm birth and baseline metabolic health status will be assessed using GLMM. These models will again be unadjusted other than treatment assignment as a covariate and adjusted for

maternal age at enrollment, race/ethnicity, and enrollment BMI. Summary statistics for all groups will be calculated as percentages.

4.3.2 Pregnancy Outcomes by Metabolic Health Status and Randomization Assignment

An interaction term will be added into the model to assess the interrelationship between health status and assignment to lifestyle intervention versus standard of care on the potential pregnancy outcomes. The models examined will be the same otherwise. Summary statistics for all groups will be calculated as percentages.

4.4 Maternal Metabolic Risk

4.4.1 Metabolic Risk Outcomes by Metabolic Health Status

The relationship changes in the metabolic risk parameters of triglycerides, total cholesterol, glucose, insulin, HDL-c, LDL-c, systolic blood pressure and diastolic blood pressure and baseline metabolic health status will be assessed using GLMM. These models will again be unadjusted other than treatment assignment as a covariate and adjusted for maternal age at enrollment, race/ethnicity, and enrollment BMI. Summary statistics for all groups will be calculated as mean ± SE at baseline (early pregnancy) and near delivery (late pregnancy).

4.4.2 Metabolic Risk Outcomes by Metabolic Health Status and Randomization Assignment

An interaction term will be added into the model to assess the interrelationship between health status and assignment to lifestyle intervention versus standard of care on the metabolic risk outcomes. The models examined will be the same otherwise. Summary statistics for all groups will be calculated as mean ± SE for the percentage change from baseline.

4.5 Neonatal Outcomes

4.5.1 Neonatal Outcomes by Metabolic Health Status

The relationship changes in the neonatal outcomes of birth weight, various skinfold measures, sum of the skinfold measures, fat mass, and fat mass index and baseline metabolic health status will be assessed using GLMM. These models will again be unadjusted other than treatment assignment as a covariate and adjusted for maternal age at enrollment, race/ethnicity, enrollment BMI, gestational age at delivery, infant sex, and infant age in days at measurement. Summary statistics for all groups will be calculated as means ± SD.

4.5.2 Metabolic Risk Outcomes by Metabolic Health Status and Randomization Assignment

An interaction term will be added into the model to assess the interrelationship between health status and assignment to lifestyle intervention versus standard of care on the metabolic risk outcomes. The models examined will be the same otherwise. Summary statistics for all groups will be calculated as means ± SD.

5 General Statistical Considerations

All analysis will be conducted utilizing SAS version 9.4. As this was not an analysis of the impact of the intervention, all participants eligible for inclusion were included in the analysis using the maximal amount of data for each outcome possible but not all participants contributed to all outcomes.

References

1. Catalano PM, Thomas AJ, Avallone DA, Amini SB. Anthropometric estimation of neonatal body composition. Am J Obstet Gynecol. 1995 Oct;173(4):1176-81. doi: 10.1016/0002-9378(95)91348-3. PMID: 7485315.
2. Grundy SM, Cleeman JI, Daniels SR, Donato KA, Eckel RH, Franklin BA, Gordon DJ, Krauss RM, Savage PJ, Smith SC Jr, Spertus JA, Costa F; American Heart Association; National Heart, Lung, and Blood Institute. Diagnosis and management of the metabolic syndrome: an American Heart Association/National Heart, Lung, and Blood Institute Scientific Statement. Circulation. 2005 Oct 25;112(17):2735-52. doi: 10.1161/CIRCULATIONAHA.105.169404. Epub 2005 Sep 12. Erratum in: Circulation. 2005 Oct 25;112(17):e297. Erratum in: Circulation. 2005 Oct 25;112(17):e298. PMID: 16157765.
