# Supplementary material for: Metabolic Health and Heterogenous Outcomes of Prenatal Interventions: A Secondary Analysis of a Randomized Clinical Trial
Source: JAMA Netw Open. 2025 Aug 21;8(8):e2528264. doi: 10.1001/jamanetworkopen.2025.28264 (PMC12371516; doi:10.1001/jamanetworkopen.2025.28264)
Supplement: Supplement 2. — eTable 1. Interventions Included in the LIFE-Moms Consortium Trial to Reduce Gestational Weight Gain eTable 2. Attainment to the 2009 NAM GWG by Obesity Phenotype and Randomized Group eTable 3. Infant Outcomes by Obesity Phenotype and Randomized Group eFigure 1. Criteria for Metabolic Health Phenotype Classification eFigure 2. Changes in Maternal Substrates and Blood Pressures Through Pregnancy [file jamanetwopen-e2528264-s002.pdf]

Supplementary Online Content

Flanagan EW, Drews KL, Cade WT, et al. Metabolic health and heterogenous outcomes of prenatal interventions: a secondary analysis of a randomized clinical trial. *JAMA Netw Open.* 2025;8(8):e2528264. doi:10.1001/jamanetworkopen.2025.28264

eTable 1. Interventions Included in the LIFE-Moms Consortium Trial to Reduce Gestational Weight Gain

eTable 2. Attainment to the 2009 NAM GWG by Obesity Phenotype and Randomized Group

eTable 3. Infant Outcomes by Obesity Phenotype and Randomized Group

eFigure 1. Criteria for Metabolic Health Phenotype Classification

eFigure 2. Changes in Maternal Substrates and Blood Pressures Through Pregnancy

This supplementary material has been provided by the authors to give readers additional information about their work.

© 2025 Flanagan EW et al. *JAMA Network Open.*

eTable 1. Interventions Included in the LIFE-Moms Consortium Trial to Reduce Gestational Weight Gain

Intervention Name	Site, Location	Trial Primary Outcome	Intervention Overview
Healthy Beginnings	California Polytechnic State University & Brown University (California, USA & Rhode Island, USA)	GWG per week	Individual counseling session (2x month from enrollment through 20 wks GA; 1–2 month from 20 wks to delivery). IBT included meal replacement product; weight graphing, weekly behavior change materials, physical activity counseling
Lifestyle Intervention for Two (LIFT)	St. Luke's – Roosevelt Hospital and Columbia University (New York, USA)	Infant percent body fat subsequent to controlling GWG	Individual counseling sessions (biweekly in-person on diet modification & physical activity counseling, with behavioral modification and social support strategies); Bolstered by weekly phone/e-mail contacts; IBT included group classes every 8 wks.
Pregnancy and EaRly Life Improvement Study (PEARLS)	University of Puerto Rico (Puerto Rico)	GWG per week below or above 2009 IOM/NAM guidelines	Counseling sessions included 2 individual and 7 group sessions. IBT was bolstered by monthly calls for improving dietary carbohydrate & fat quality and physical activity counseling.
Maternal Offspring Metabolics: Family Intervention Trial (MOMFIT)	Northwestern University (Illinois, USA)	Total GWG	Counseling sessions included 3 individual and 6 group sessions with phone coaching on diet and physical activity. IBT was bolstered by weekly electronic feedback from participant self-monitoring using the "Loseit!" app
PreGO	Washington University (Missouri, USA)	Total GWG exceeding IOM/NAM guidelines	10 Parents as Teachers (PAT) home visits incorporating diet and physical activity focusing on control of weight gain reflecting the PAT philosophy.
Expecting Success	Pennington Biomedical Research Center (Louisiana, USA)	GWG per week exceeding IOM guidelines	Two groups (clinic vs phone) that promoted similar dietary intake (55% carbs, 15% protein, 30% fat) and exercise counseling through 18 sessions that were focused on appropriate weight gain. Clinic group received IBT through individual and group sessions while the phone group received feedback from weight and physical activity data transmitted using Bluetooth devices.
LIFE-Moms Pheonix	NIDDK/Pheonix Indian Medical Center (Arizona, USA)	Total GWG	Weekly group or individual sessions focused on individualized managed weight gain goals through caloric and fat gram recommendations, physical activity, decreased sedentary time.

GWG: gestational weight gain; GA: gestational age; IBT: Intensive behavioral therapy; NIDDK: National Institute of Diabetes and Digestive and Kidney Diseases.

© 2025 Flanagan EW et al. *JAMA Network Open.*

eTable 2. Attainment to the 2009 NAM GWG by Obesity Phenotype and Randomized Group

	**Metabolically Healthy**		**Metabolically Unhealthy**		
	Treatment	Control	Treatment	Control	
	**% ± SE**	**% ± SE**	**% ± SE**	**% ± SE**	**p-value (interaction)**
**Attainment to the 2009 NAM GWG guidelines**					
Below	23.3 ± 3.9	14.3 ± 3.4	34.7 ± 5.5	23.4 ± 4.4	0.59
Within	17.5 ± 3.5	6.7 ± 2.4	22.7 ± 4.9	8.5 ± 2.9	0.84
Excess	59.2 ± 4.5	79.0 ± 4.0	42.7 ± 5.7	68.1 ± 4.8	0.88

NAM: National Academy of Medicine; GWG: gestational weight gain

© 2025 Flanagan EW et al. *JAMA Network Open.*

eTable 3. Infant Outcomes by Obesity Phenotype and Randomized Group

	**Metabolically Healthy**		**Metabolically Unhealthy**		
	Treatment **Mean ± SE**	Control **Mean ± SE**	Treatment **Mean ± SE**	Control **Mean ± SE**	**p-value (interaction)**
**Infant Outcomes**					
Birth weight (kg)	3.22 ± 0.062	3.20 ± 0.062	3.34 ± 0.070	3.24 ± 0.064	0.42
Subscapular skinfold thickness (mm)	4.87 ± 0.15	4.70 ± 0.16	5.17 ± 0.17	4.95 ± 0.16	0.84
Sum of skinfold thicknesses (mm)	20.71 ± 0.62	20.71 ± 0.63	22.16 ± 0.69	21.47 ± 0.65	0.48
Neonatal fat (%)	11.86 ± 0.42	11.22 ± 0.43	12.65 ± 0.52	12.18 ± 0.45	0.85

Models adjusted for maternal baseline age and BMI, infant sex, gestational age at delivery, and number of days between delivery and neonatal measurements. BMI: body mass index; kg: kilograms; mm: millimeters

© 2025 Flanagan EW et al. *JAMA Network Open.*

eFigure 1. Criteria for Metabolic Health Phenotype Classification

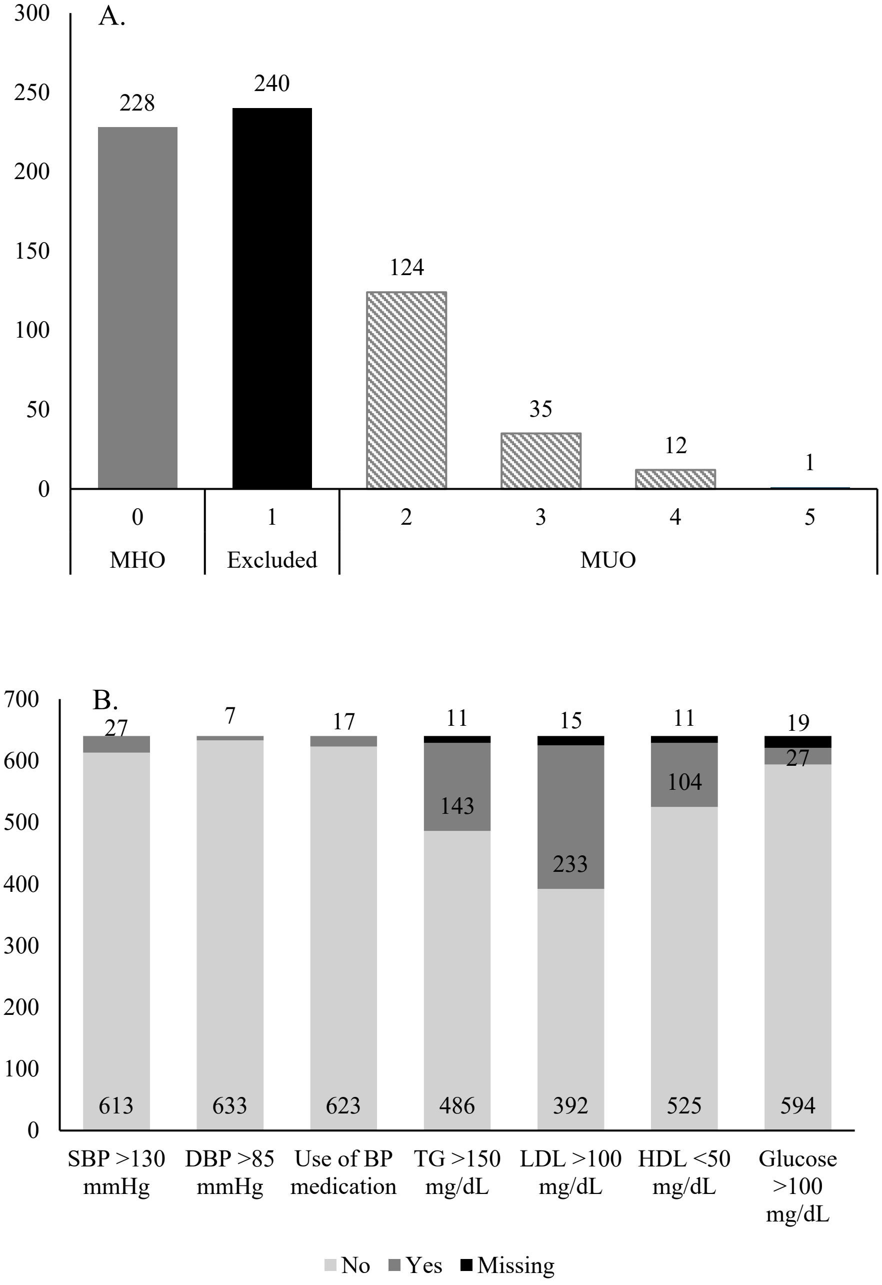

(A) Number of ATP III criteria fulfilled alongside obesity and (B) Number of participants with underlying conditions. MHO: Metabolically Healthy Obesity; MUO: Metabolically Unhealthy Obesity; SBP: Systolic blood pressure; DBP: Diastolic blood pressure; BP: blood pressure; TG: triglycerides; LDL: low-density lipoprotein; HDL: high-density lipoprotein.

© 2025 Flanagan EW et al. *JAMA Network Open.*

eFigure 2. Changes in Maternal Substrates and Blood Pressures Through Pregnancy

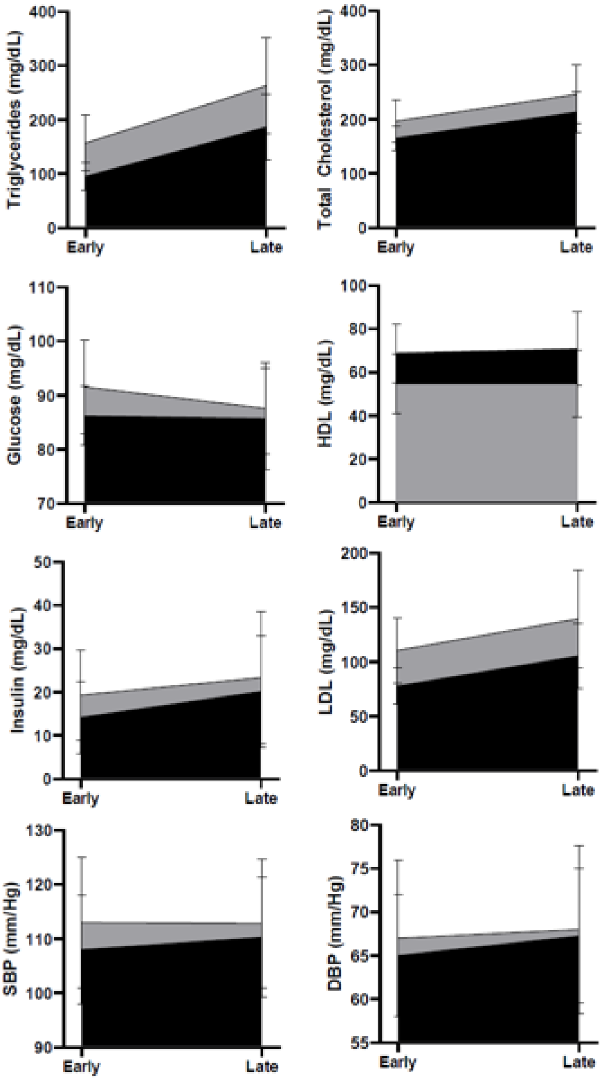

© 2025 Flanagan EW et al. *JAMA Network Open.*
