# Supplementary material for: Metabolic Health and Heterogenous Outcomes of Prenatal Interventions: A Secondary Analysis of a Randomized Clinical Trial
Source: JAMA Netw Open. 2025 Aug 21;8(8):e2528264. doi: 10.1001/jamanetworkopen.2025.28264 (PMC12371516; doi:10.1001/jamanetworkopen.2025.28264)
Supplement: Supplement 3. — Data Sharing Statement [file jamanetwopen-e2528264-s003.pdf]

Data Sharing Statement

Flanagan. Metabolic Health and Heterogenous Outcomes of Prenatal Interventions. *JAMA Netw Open*. Published August 21, 2025. doi:10.1001/jamanetworkopen.2025.28264

Data
Additional Information: Registered on Clinicaltrials.gov as NCT01545934, NCT01616147, NCT01771133, NCT01631747, NCT01768793, NCT01610752, and NCT01812694
Data available: Yes
Data types: Deidentified participant data, Data dictionary
How to access data: Lifestyle Interventions For Expectant Mothers (LIFE-Moms) was conducted by the LIFE-Moms Research Group and supported by the National Institute of Diabetes and Digestive and Kidney Diseases (NIDDK), the National Heart, Lung, and Blood Institute (NHLBI), the Eunice Kennedy Shriver National Institute of Child Health and Human Development (NICHD), the National Center for Complementary and Integrative Health (NCCIH), the NIH Office of Research in Women's Health (ORWH), the Office of Behavioral and Social Science Research (OBSSR), the NIH Office of Disease Prevention (ODP), the Indian Health Service, the Intramural Research Program of the NIDDK, and the Office of the Director, National Institutes of Health (OD). The data from LIFE-Moms are available at the NIDDK Central Repository (NIDDK-CR), Resources for Research (R4R) at https://repository.niddk.nih.gov/studies/life-moms/.
When available: beginning date: 03-02-2023

Supporting Documents
Document types: Other (please specify)
Additional Information: Protocol, data dictionary
How to access documents: https://repository.niddk.nih.gov/studies/life-moms/
When available: beginning date: 03-02-2023

Additional Information
Who can access the data: Researchers whose proposed use of the data has been approved
Types of analyses: Any scientific analyses, within reason
Mechanisms of data availability: after approval of a proposal
